# Racial Disparities in Survival Outcomes for Metastatic Renal Cell Carcinoma with Sarcomatoid Differentiation: A SEER-Based Retrospective Analysis (2010–2020)

**DOI:** 10.3390/cancers18172884

**Published:** 2026-09-06

**Authors:** Lingbin Meng, Xiaowei Malone, Hui Peng, Lin Mei, Changchuan Jiang, Xuefeng Liu, Qi-En Wang, Feng Hong, Akshay Sood, Shang-Jui Wang, Qingqing Wu, Shihua Wang, Peng Wang

**Affiliations:** 1 Division of Medical Oncology, Department of Internal Medicine, The Ohio State University Comprehensive Cancer Center, Columbus, OH 43210, USA; lingbin.meng@osumc.edu (L.M.); hui.peng@osumc.edu (H.P.); feng.hong@osumc.edu (F.H.); 2 AdventHealth, Tampa, FL 33613, USA; xiaowei.malone.do@adventhealth.com; 3 Department of Hematology and Oncology, University of Pennsylvania, Philadelphia, PA 19104, USA; lin.mei@pennmedicine.upenn.edu; 4 Department of Internal Medicine, University of Texas Southwestern Medical Center, Dallas, TX 75390, USA; changchuan.jiang@utsouthwestern.edu; 5 Department of Pathology, The Ohio State University Comprehensive Cancer Center, Columbus, OH 43210, USA; xuefeng.liu@osumc.edu (X.L.); qingqing.wu@osumc.edu (Q.W.); 6 Department of Radiation Oncology, The Ohio State University Comprehensive Cancer Center, Columbus, OH 43210, USA; qi-en.wang@osumc.edu (Q.-E.W.); shang-jui.wang@osumc.edu (S.-J.W.); 7 Department of Urology, The Ohio State University Wexner Medical Center, Columbus, OH 43210, USA; akshay.sood@osumc.edu

**Keywords:** renal cell carcinoma, sarcomatoid, metastatic, race and ethnicity, mortality, clear cell

## Abstract

**Background:** Sarcomatoid renal cell carcinoma (sRCC) is an aggressive RCC subtype with a poor prognosis. Although racial disparities in RCC outcomes are reported, survival patterns across racial and ethnic groups in metastatic sRCC (msRCC) remain unclear. **Methods:** We conducted a retrospective analysis of 2122 patients with msRCC diagnosed between 2010 and 2020 using the Surveillance, Epidemiology, and End Results (SEER) database. Patients were categorized by race and ethnicity as non-Hispanic White, non-Hispanic Black, Hispanic, Asian/Pacific Islander, or American Indian/Alaska Native. Three-year cancer-specific survival (CSS) and overall survival (OS) were estimated using Kaplan–Meier methods. Multivariable Cox proportional hazards regression models were used to evaluate racial disparities in survival. **Results:** Non-Hispanic Black patients experienced the poorest outcomes, with 3-year CSS and OS of 9.3% and 8.6%, compared with 23.3% and 20.9% in non-Hispanic White, 22.1% and 19.0% in Hispanic, 21.2% and 19.4% in Asian/Pacific Islander, and 23.4% and 20.7% in American Indian/Alaska Native patients. Non-Hispanic Black patients were associated with higher estimated hazards of cancer-specific mortality (HR = 1.5, *p* < 0.0001) and overall mortality (HR = 1.5, *p* < 0.0001) versus non-Hispanic White. From 2010–2015 to 2016–2020, survival improved across most racial groups with the introduction of immune checkpoint inhibitors. However, non-Hispanic Black patients remained the only group with persistently inferior survival. Subgroup analysis further demonstrated worse survival for non-Hispanic Black patients in both clear cell msRCC and non-clear cell msRCC subgroups. **Conclusions:** Although survival was higher during the later diagnosis period, non-Hispanic Black patients with msRCC continued to have the poorest observed survival. These differences may be associated with unmeasured social, structural, socioeconomic, healthcare access, treatment, or clinical factors, but their causes cannot be determined from this observational study. Further research is needed to identify the factors underlying these survival differences.

## Simple Summary

Sarcomatoid renal cell carcinoma is a rare and aggressive form of kidney cancer that often spreads and leads to poor outcomes. Previous research has shown that survival can differ among racial and ethnic groups in kidney cancer, but it is not well understood whether these differences exist in this particularly severe form. This study aims to examine associations between race and ethnicity and survival using a large national database. The authors seek to identify whether certain groups face a higher estimated hazard of death and whether outcomes have improved over time with newer treatments. The findings highlight ongoing differences in survival, especially for Black patients, and suggest that factors such as access to care, social conditions, or biological differences may play a role. This work may help guide future research and efforts to reduce disparities in cancer outcomes.

## 1. Introduction

Renal cell carcinoma (RCC) accounts for approximately 90% of all kidney cancers. Clear cell RCC (ccRCC) is the most common histologic subtype, comprising about 75% of cases, followed by papillary (15%), chromophobe (5%), collecting duct (1%), renal medullary carcinoma, MiT family translocation, mucinous tubular and spindle cell, and unclassified RCC [1,2]. Sarcomatoid dedifferentiation is a rare but aggressive transformation that can occur across most RCC histologic subtypes. Tumors exhibiting this feature are commonly referred to as sarcomatoid RCC (sRCC).

sRCC is associated with an especially poor prognosis, with 60–80% of patients presenting with advanced or metastatic disease at diagnosis. Historically, conventional therapies have demonstrated limited efficacy in this population, with reported median survival ranging from 6 to 13 months [1,2]. Given its rarity and advanced stage at presentation, sRCC has been underrepresented in clinical studies, resulting in limited data to guide management.

Racial disparities have been observed in RCC across multiple domains, including incidence, stage at diagnosis, treatment patterns, and survival outcomes [3,4,5]. Black patients are more likely to be diagnosed with locally advanced disease and are less likely to receive nephrectomy compared to White patients [4,5]. Overall survival (OS) is often worse among Black patients, largely attributable to delayed diagnosis and disparities in access to timely, high-quality care [6,7,8]. However, the impact of racial disparities on patients with msRCC remains largely unexplored. Understanding these disparities within this high-risk subset is essential to inform equitable treatment strategies.

Immune checkpoint inhibitors (ICIs) have transformed the treatment landscape for advanced and metastatic RCC. Prior studies have demonstrated that sRCCs exhibit higher expression of programmed cell death ligand 1 (PD-L1) on tumor cells and programmed cell death protein 1 (PD-1) on tumor-infiltrating lymphocytes compared with non-sarcomatoid RCCs, suggesting improved responses to PD-1- and PD-L1-blocking agents. Consequently, ICIs represent a promising therapeutic strategy for sRCC. The introduction of ICI-based regimens, including nivolumab plus ipilimumab and pembrolizumab plus axitinib, has led to improved outcomes in patients with msRCC; nevertheless, this remains the most aggressive RCC subtype with persistently poor survival [9,10,11,12].

In this study, we used the Surveillance, Epidemiology, and End Results (SEER) database to evaluate 3-year cancer-specific survival (CSS) and OS among 2122 patients diagnosed with msRCC between 2010 and 2020. We examined racial disparities across non-Hispanic White, non-Hispanic Black, Hispanic, Asian/Pacific Islander, and American Indian/Alaska Native populations and assessed changes in survival patterns before and after the widespread adoption of ICIs (2010–2015 vs. 2016–2020). Additionally, we determined outcomes among patients with clear cell and non-clear cell histologies. To our knowledge, this represents the largest retrospective analysis of racial disparities in msRCC, providing critical insights to support the development of more equitable cancer care.

## 2. Methods

Patients diagnosed with RCC were identified from the SEER 17 Registries Research Data F(November 2022 submission, 2000–2020) using SEER*Stat version 8.4.2 (accessed in July 12, 2023) and the primary site code C64.9. Sarcomatoid differentiation was defined using the SarcomatoidFeatures_Recode_2010 variable, which is characterized by sheets and fascicles of malignant spindle cells and may occur across all RCC histologic subtypes. As this variable is only available for cases diagnosed in 2010 or later, the study cohort was restricted to patients diagnosed between 2010 and 2020. The administrative censoring date was 31 December 2020, for the entire cohort. Only patients with msRCC classified as M1 under the TNM staging system were included. Because SEER primarily records disease extent at diagnosis, our cohort represents patients with de novo metastatic sRCC. Patients with unknown data on survival time, race, and ethnicity were excluded from the analysis. The patient-selection process is illustrated in Supplementary Figure S1.

Additional covariates obtained from the SEER database included sex, age at diagnosis, marital status, and median household income, which was inflation-adjusted to 2021 US dollars and hereafter referred to as “income.” It represents a geographically aggregated measure of median household income rather than an individual patient’s earnings. Race and ethnicity were categorized using the race and ethnicity variable into the following groups: non-Hispanic White (hereafter referred to as White), non-Hispanic Black (hereafter referred to as Black), Hispanic (all races), non-Hispanic Asian or Pacific Islander (hereafter referred to as Asian or Pacific Islander), and non-Hispanic American Indian/Alaska Native (hereafter referred to as American Indian/Alaska Native).

Histologic subtypes were classified according to the International Classification of Diseases for Oncology, Third Edition (ICD-O-3) codes as follows: clear cell carcinoma (8005, 8010,8140, 8310, 8312, 8318, 8320, and 8323), papillary carcinoma (8050 and 8260), chromophobe (8317 and 8270), and other histology [13]. The number of patients associated with each ICD-O-3 code and the corresponding histologic subtype are presented in Supplementary Table S1. Additional clinical variables included T and N stage, tumor grade (I–IV or unknown), and treatment modalities, including surgery, systemic therapy, and radiation therapy. Those who received surgery were defined by “Surgery performed” in the variable “Reason no cancer-directed surgery”. Radiotherapy was identified by “Beam radiation”, “Combination of beam with implants or isotopes”, “Radiation, NOS method or source not specified”, “Radioactive implants (includes brachytherapy) (1988+)”, and “Radioisotopes (1988+) in the variable “Radiation_recode(Radiation recode)”. Systemic therapy was identified by “Yes” in the variable “Chemotherapy recode (yes, no/unk)”.

### Statistical Analysis

Continuous variables were summarized as means ± standard deviations and compared across racial and ethnic groups using analysis of variance (ANOVA). When overall differences were significant, post hoc pairwise comparisons were performed using Tukey’s test. Categorical variables were summarized as frequencies and percentages and compared using the chi-square test.

Median follow-up was estimated using the reverse Kaplan–Meier method, in which deaths were treated as censored observations and patients censored in the conventional survival analysis were treated as events. Kaplan–Meier survival curves were generated to estimate 3-year CSS and OS, and differences between groups were assessed using the log-rank test. Cumulative incidence functions for cancer-specific mortality were plotted by racial group, treating deaths from other causes as competing events. We assessed the proportional-hazards assumption for all major variables by testing interactions between each covariate and log-transformed survival time. None of these interactions was statistically significant, indicating no evidence that the proportional-hazards assumption was violated. Univariable and multivariable Cox proportional hazards regression models were used to estimate hazard ratios (HRs) and corresponding 95% confidence intervals (CIs) for cancer-specific and overall mortality. Variables with *p* < 0.20 in univariable analysis were considered for inclusion in the multivariable survival models. Forward selection was then applied, and variables with *p* < 0.10 were retained in the final models. The primary estimand was the covariate-adjusted cause-specific hazard ratio for cancer-specific mortality comparing each racial/ethnic group with non-Hispanic White patients among individuals with de novo msRCC, measured from diagnosis until cancer-specific death or censoring. Overall survival was evaluated using the covariate-adjusted all-cause hazard ratio as a secondary estimand. Analyses stratified by diagnosis period and histologic subtype were considered secondary and exploratory. For comparisons of 3-year survival between the 2010–2015 and 2016–2020 diagnosis periods, we conducted a sensitivity analysis restricting the later-period cohort to patients diagnosed by 31 December 2017, ensuring the potential for at least three years of follow-up for all included patients.

All statistical analyses were performed using SAS version 9.4 (SAS Institute Inc., Cary, NC, USA). A 2-sided *p* value < 0.05 was considered statistically significant.

## 3. Results

### 3.1. Characteristics of Patients with msRCC

A total of 2122 patients with msRCC were identified from the SEER database, of whom 67.9% were White, 8.0% Black, 17.2% Hispanic, 5.5% Asian and Pacific Islander, and 1.4% American Indian/Alaska Native. The clinicopathologic characteristics of patients between 2010 and 2020 are summarized in Table 1. There were significant differences in most sociodemographic and clinical characteristics by race and ethnicity.

Overall, patients were predominantly aged 60–69 years (34.3%), with fewer patients aged <50 (11.7%) or ≥80 (5.9%). White patients were more likely to be aged 60–69 or 70–79 years, whereas Hispanic and Black patients had a higher proportion in younger age groups (< 50 and 50–59 years) (*p* < 0.0001). The cohort was mostly male (70.9%), with a slightly higher proportion of males among White patients and higher proportions of females among Hispanic and Asian and Pacific Islander patients (*p* = 0.0121).

Most patients were married (63.4%), but marital status varied by race and ethnicity; White and Asian and Pacific Islander patients were more likely to be married, whereas Black and American Indian/Alaska Native patients had higher proportions of unmarried individuals (*p* < 0.0001). Regarding geographic region, more than half of the cohort resided in the West (53.4%), followed by the South (27.7%), Northeast (13.8%), and Midwest (5.2%). Hispanic and Asian and Pacific Islander patients were predominantly from the West, whereas White and Black patients were more commonly from the South and Northeast (*p* < 0.0001).

Income distribution was roughly even overall, with 27.6% of patients residing in areas with an income of <$60,000, 36.2% in areas with an income of $60,000–$74,999, and 36.2% in areas with an income of ≥$75,000. White and Black patients were more likely to be in the lower-income areas, whereas Asian and Pacific Islander patients were more likely to reside in areas with incomes of ≥$75,000 (*p* < 0.0001).

With respect to tumor characteristics, most patients had T3 disease (51.9%), followed by T4 (19.3%), T2 (11.4%), and T1 (10.2%), with significant racial and ethnic differences in T-stage distribution (*p* = 0.0025). Nodal status did not significantly differ across groups (*p* = 0.3641), with the majority being N0 (56.1%). Most tumors were >3 cm (91.3%), with no significant racial and ethnic differences noted in patient tumor size (*p* = 0.3282).

Clear cell histology was the most common subtype (82.0%), followed by “other” histologies (14.1%), with smaller proportions of papillary (2.7%) and chromophobe (1.2%); histology distribution differed by race and ethnicity (*p* = 0.0042). Tumor grade did not significantly vary across groups (*p* = 0.1428).

Regarding treatment, most patients underwent surgery (71.3%), although Black patients were less likely to receive surgery compared with other groups (*p* = 0.0293). Approximately half of patients received systemic therapy (49.9%), with no significant differences seen based on race and ethnicity (*p* = 0.7514). Radiotherapy was used in 27.9% of patients with similar proportions across groups (*p* = 0.4818).

By the end of the study period, 72.0% of patients experienced cancer-specific death, and 78.0% experienced overall death.

Median follow-up, estimated using the reverse Kaplan–Meier method, was 58 months (95% CI, 56–60) overall, 76 months (95% CI, 73–79) for patients diagnosed in 2010–2015, and 31 months (95% CI, 29–33) for those diagnosed in 2016–2020.

### 3.2. Black Patients Had the Worst Observed Survival Outcome Among Racial Groups in msRCC

**Figure 1:**
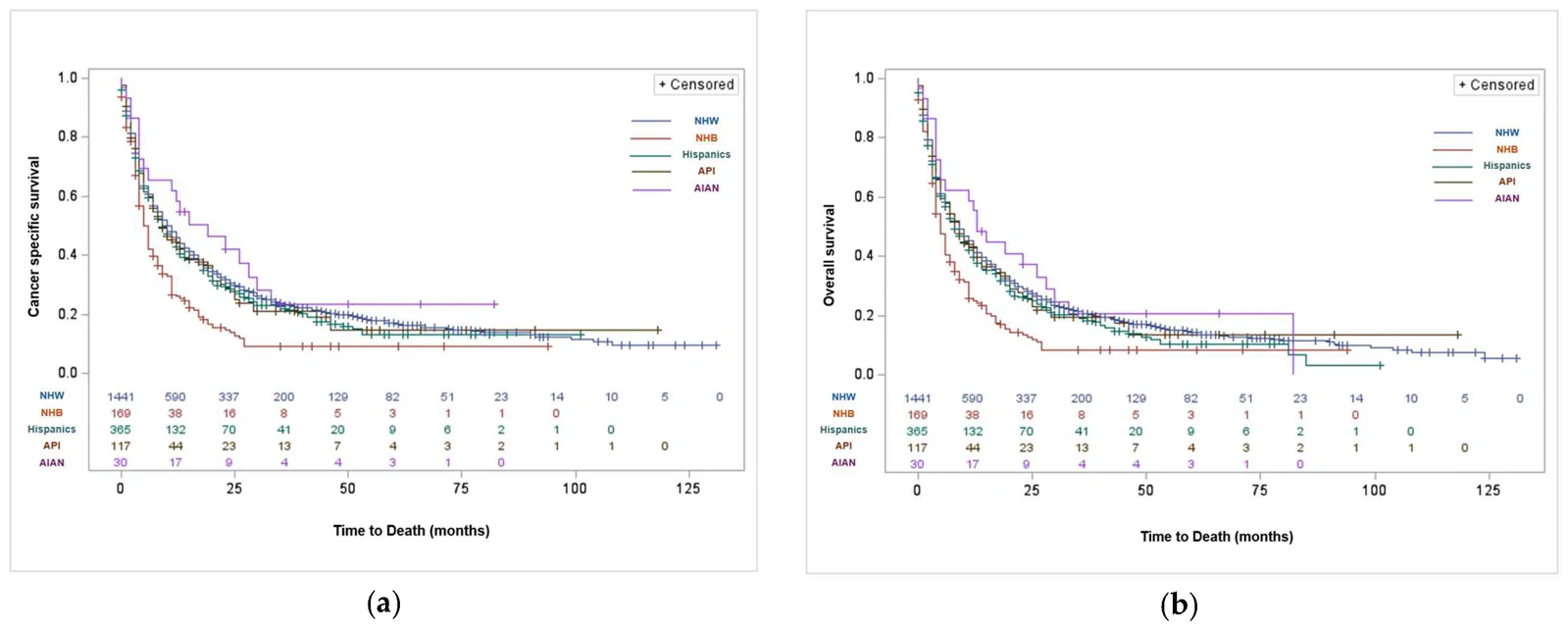
Cancer-specific survival (**a**) and overall survival (**b**) of different racial groups during 2010–2020. AIAN, Non-Hispanic American Indian and Alaska Native; API, Non-Hispanic Asian or Pacific Islanders; NHB, Non-Hispanic Black; NHW, Non-Hispanic White.

Three-year CSS and OS differed significantly across racial and ethnic groups (Figure 1a,b and Supplementary Table S2). Black patients demonstrated the poorest outcomes, with a 3-year CSS of 9.3% compared with 23.3% among White patients. Similarly, 3-year OS was significantly lower in Black patients than in White patients (8.6% vs. 20.9%). No significant differences in 3-year CSS or OS were observed between White patients and other racial and ethnic groups. Non-Hispanic Black patients clearly showed the highest cumulative incidence of cancer-specific mortality (Supplementary Figure S2). This pattern of racial disparities in survival was consistently observed in the univariate Cox proportional hazards regression analyses (Supplementary Table S2).

### 3.3. Multivariable Analysis of Factors Associated with Survival in msRCC

Multivariable Cox regression analyses (Table 2) showed that, compared with cc msRCC, chromophobe msRCC was associated with significantly worse CSS (HR = 1.8, *p* = 0.0167) and OS (HR = 1.8, *p* = 0.008), while papillary msRCC exhibited no significance in CSS (*p* = 0.3891) and OS (*p* = 0.3876).

Age was independently associated with survival outcomes. Patients aged 50–59 years demonstrated improved OS compared with those <50 years (HR = 0.8, *p* = 0.0282), although no significant difference in CSS was observed. In contrast, patients aged ≥ 80 years experienced significantly worse CSS (HR = 1.7) and OS (HR = 1.9) compared with those < 50 years (both *p* < 0.0001).

Race and ethnicity remained an independent predictor of survival. Black patients were associated with significantly higher estimated hazards of CSS (HR = 1.5, 95% CI: 1.3–1.8, *p* < 0.0001) and OS (HR = 1.5, 95% CI: 1.2–1.7, *p* < 0.0001) compared with White patients. Hispanic patients also experienced inferior OS relative to White patients (HR = 1.2, 95% CI: 1.0–1.3, *p* = 0.0249), although no significant difference in CSS was observed. Survival outcomes for Asian and Pacific Islander and American Indian/Alaska Native patients did not differ significantly from those of White patients.

Socioeconomic status was significantly associated with survival, as patients with area-level income ≥ $75,000 demonstrated a lower estimated hazard of CSS (HR = 0.9, 95% CI: 0.8–1.0, *p* = 0.0177) and OS (HR = 0.8, 95% CI: 0.7–1.0, *p* = 0.0068). Tumor stage was a strong prognostic factor, with advanced T stage (T4) and nodal involvement (N1) associated with significantly worse CSS and OS (both *p* < 0.0001).

Treatment-related variables were also significant predictors of outcome. Patients who underwent surgery experienced significantly lower estimated hazard of CSS and OS (both HR = 0.4, *p* < 0.0001), and receipt of systemic therapy was associated with better CSS and OS (both HR = 0.7, *p* < 0.0001). In contrast, receipt of radiotherapy was associated with inferior outcomes, including a 20% increase in cancer-specific mortality (HR = 1.2, *p* = 0.0109) and a 10% increase in overall mortality (HR = 1.1, *p* = 0.0349).

### 3.4. Black Patients Remained the Only Group with Significantly Worse Survival After the Introduction of ICIs

To evaluate whether recent therapeutic advances have altered the racial disparities observed in msRCC, we conducted a stratified analysis comparing patients diagnosed in 2010–2015 with those diagnosed in 2016–2020. A total of 1080 patients were diagnosed in 2010–2015 and 1042 patients in 2016–2020. Patient demographics and baseline characteristics for each period are summarized in Table 3 and Table 4.

Across both cohorts, non-White patients were diagnosed at younger ages (< 70 years) compared with White patients. Black patients were consistently the least likely to undergo surgery (58.6% in 2010–2015 vs. 63.4% in 2016–2020), suggesting persistent disparities in access to or eligibility for surgical treatment. Socioeconomic disparities not only persisted but appeared to widen over time. The proportion of White patients living in the highest-income areas increased from 29.0% to 41.3%, whereas the proportion of Black patients living in the lowest-income areas rose from 32.2% to 34.2%. Additionally, a higher proportion of Black and Hispanic patients remained unmarried in both cohorts, with this disparity becoming more pronounced in the later period; for example, the proportion of unmarried Black patients increased from 43.7% to 59.8%, potentially reflecting diminished social support.

Regional distribution remained relatively stable over time, with Black patients predominantly residing in the Southern region and other racial groups concentrated in the Western region. Regarding tumor characteristics at presentation, Black patients continued to have the highest proportion of T1 tumors, while Hispanic patients had the highest proportion of T4 tumors. Notably, during the 2010–2015 period, Black patients had a significantly higher proportion of non-clear cell histology compared with White patients; however, this difference was no longer observed in the 2016–2020 cohort, suggesting a shift in histologic distribution or diagnostic patterns. We also noted that the proportion of grade III/IV tumors was substantially higher among patients diagnosed in 2010–2015 than among those diagnosed in 2016–2020 (70.5% vs. 36.5%). However, grade III/IV and unknown-grade tumors combined accounted for more than 98% and 97% of cases during the two respective periods.

**Figure 2:**
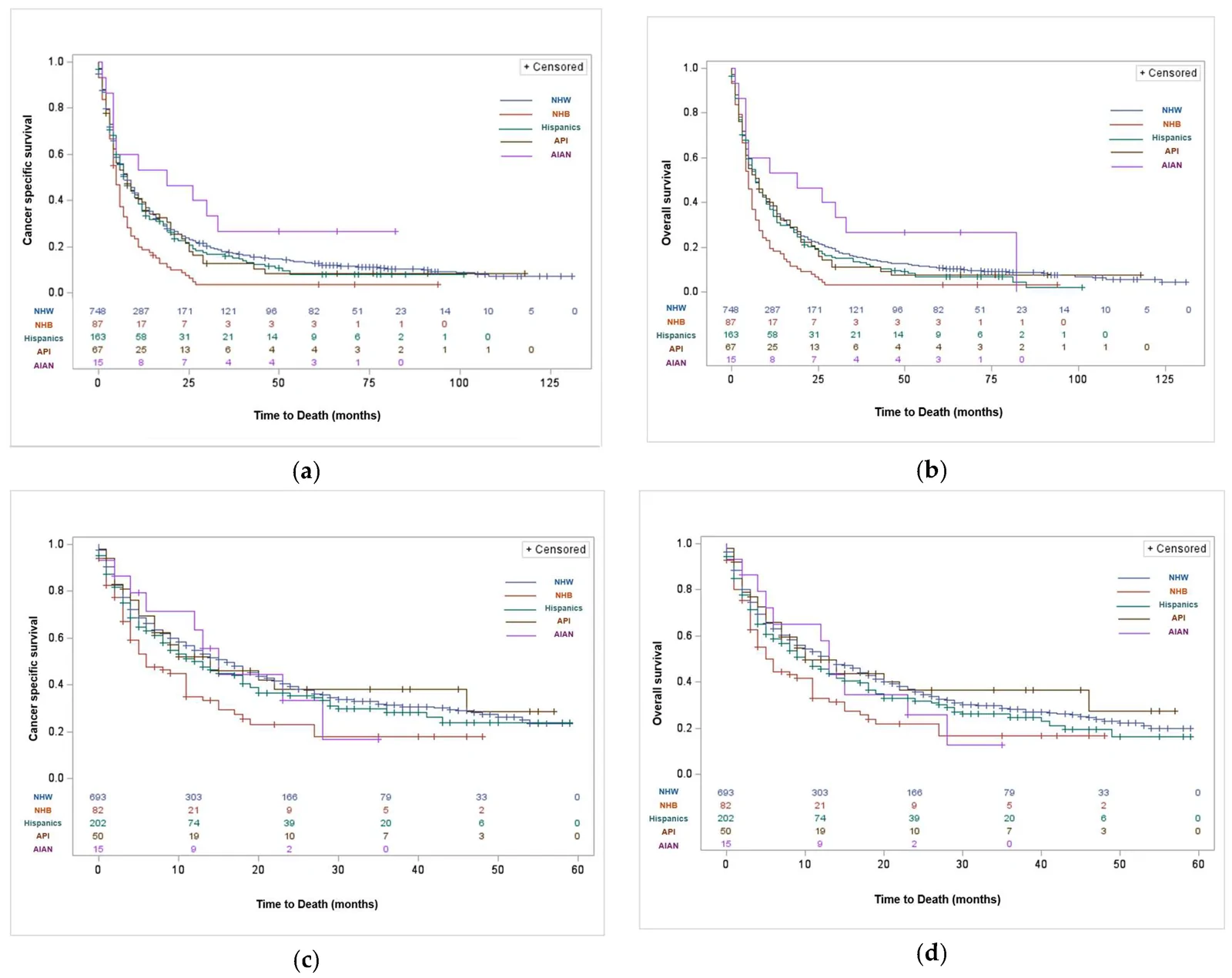
Cancer-specific survival and overall survival of different racial groups during 2010–2015 vs. 2016–2020. (**a**) Three-year cancer-specific survival during 2010–2015; (**b**) overall survival during 2010–2015; (**c**) three-year cancer-specific survival during 2016–2020; (**d**) overall survival during 2016–2020. AIAN, Non-Hispanic American Indian and Alaska Native; API, Non-Hispanic Asian or Pacific Islanders; NHB, Non-Hispanic Black; NHW, Non-Hispanic White.

Overall, 3-year CSS and OS improved across most racial groups from 2010–2015 to 2016–2020 (Figure 2 and Supplementary Table S2). Among all racial groups, Asian and Pacific Islander patients experienced the greatest improvement, with increases of 25.6 percentage points in CSS and 25.3 percentage points in OS. Hispanic and Asian and Pacific Islander patients demonstrated no significant differences in survival outcomes compared to those White patients in the later period, reflecting substantial gains following the introduction of ICIs. American Indian/Alaska Native patients were the only group to experience a decline in survival, although interpretation is limited by a small sample size (n = 30). Black patients also experienced improved outcomes, with increases of 14.3 percentage points in 3-year CSS and 13.4 percentage points in OS; however, they remained the only racial group with significantly worse CSS and OS compared with White patients in both time periods. Non-Hispanic Black patients likewise showed the highest cumulative incidence of cancer-specific mortality among patients diagnosed during 2010–2015 (Supplementary Figure S3) and during 2016–2010 (Supplementary Figure S4).

Multivariable survival analyses stratified by diagnosis period (Table 5) confirmed the persistence of racial disparities. The Black demographic remained independently associated with worse outcomes in both eras. In 2010–2015, Black patients had a 50% higher estimated hazard of cancer-specific mortality (HR = 1.5, 95% CI: 1.1–1.8, *p* = 0.0025) and a 40% higher estimated hazard of all-cause mortality (HR = 1.3, 95% CI: 1.1–1.7, *p* = 0.01) compared with White patients. These disparities persisted in 2016–2020, with Black patients experiencing an 80% higher estimated hazard of cancer-specific mortality (HR = 1.8, 95% CI: 1.3–2.4, *p* = 0.0002) and a 60% higher estimated hazard of overall mortality (HR = 1.6, 95% CI: 1.2–2.1, *p* = 0.0012). In a sensitivity analysis restricted to patients diagnosed during 2016–2017 to reduce the potential influence of unequal follow-up, non-Hispanic Black patients were associated with higher estimated hazards of cancer-specific mortality (HR = 1.7, 95% CI: 1.1–2.6, *p* = 0.0091) and overall mortality (HR = 1.6, 95% CI: 1.1–2.4, *p* = 0.0224) than non-Hispanic White patients (Supplementary Table S3).

Histologic subtype remained a significant predictor of survival across both periods. During 2010–2015, papillary msRCC was associated with a significantly lower estimated hazard of cancer-specific mortality (HR = 0.6, 95% CI: 0.4–0.9, *p* = 0.0256) and overall mortality (HR = 0.6, 95% CI: 0.4–0.9, *p* = 0.0214). In the 2016–2020 cohort, both chromophobe and “other” histologies were associated with significantly worse CSS and OS compared with cc msRCC.

Advanced age was consistently associated with poorer outcomes. Patients aged ≥80 years had significantly worse CSS and OS in both periods, although outcomes modestly improved in the later cohort. In 2010–2015, patients aged ≥ 80 years had a 90% higher estimated hazard of cancer-specific mortality (HR = 1.9, 95% CI: 1.3–2.6, *p* = 0.0003) and a 100% higher estimated hazard of overall mortality (HR = 2.0, 95% CI: 1.5–2.8, *p* < 0.0001). In 2016–2020, the corresponding estimated hazards decreased to 60% for both cancer-specific mortality (HR = 1.6, 95% CI: 1.1–2.3, *p* = 0.0272) and overall mortality (HR = 1.7, 95% CI: 1.2–2.4, *p* = 0.0042).

Treatment-related factors remained strongly prognostic. Surgical intervention was associated with significantly improved CSS and OS in both periods (*p* < 0.0001). Systemic therapy was also associated with improved survival, although its effect was more pronounced in the earlier cohort. In 2010–2015, systemic therapy was associated with approximately a 40% reduction in the estimated hazard of cancer-specific mortality (HR = 0.6, *p* < 0.0001) and a 50% reduction in overall mortality (HR = 0.5, *p* < 0.0001). In contrast, in 2016–2020, systemic therapy was associated with a slightly lower estimated hazard of cancer-specific mortality (HR = 0.9, *p* = 0.0785) and overall mortality (HR = 0.9, *p* = 0.0453).

Similarly, the sequential models consistently demonstrated racial disparities in cancer-specific mortality and overall mortality (Supplementary Tables S4 and S5).

### 3.5. Racial and Ethnic Differences in Survival Within Clear-Cell and Non-Clear-Cell msRCC Subgroups

Among both histologic subtypes, Black patients consistently exhibited the poorest outcomes, with the lowest CSS in cc msRCC (11.4%) and non-cc msRCC (0%), as well as the lowest OS in cc msRCC (10.8%) and non-cc msRCC (0%).

**Figure 3:**
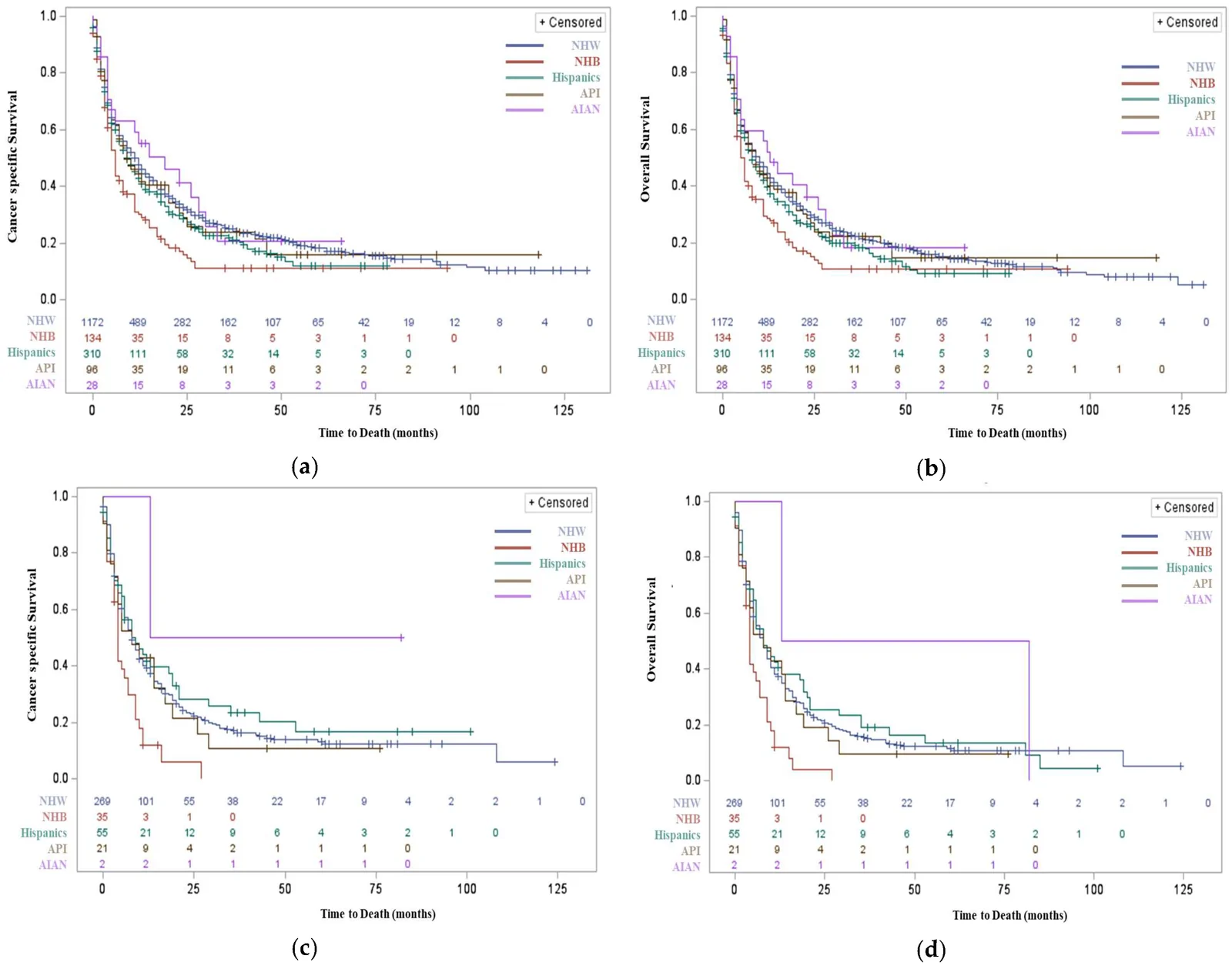
Cancer-specific survival and overall survival of different racial groups with cc msRCC vs. non-cc msRCC subtypes during 2010–2020. (**a**) Three-year cancer-specific survival of cc msRCC; (**b**) overall survival of cc msRCC; (**c**) three-year cancer-specific survival of non-cc msRCC; (**d**) overall survival of non-cc msRCC. AIAN, Non-Hispanic American Indian and Alaska Native; API, Non-Hispanic Asian or Pacific Islanders; NHB, Non-Hispanic Black; NHW, Non-Hispanic White. Cc msRCC: clear cell metastatic sarcomatoid renal cell carcinoma; non-cc msRCC: non-clear cell metastatic sarcomatoid renal cell carcinoma.

Univariable Cox regression analyses showed that, in cc msRCC, significant racial differences in CSS and OS were observed between White and Black patients (*p* < 0.0001 and 0.0003, respectively), but not between other races and White patients. Similarly, statistically significant differences in CSS and OS were observed between Black and White patients (*p* = 0.0005 and *p* = 0.0004, respectively) identified within the non-cc msRCC cohort (Figure 3 and Supplementary Table S6). Non-Hispanic Black patients likewise showed the highest cumulative incidence of cancer-specific mortality in both cc-msRCC (Supplementary Figure S4) and non-cc-msRCC (Supplementary Figure S5) subgroups.

Multivariable Cox regression analyses stratified by histology (Table 6) further supported these findings. Among patients with cc msRCC, those aged 50–59 years had significantly lower estimated hazard of cancer-specific mortality (HR = 0.8, *p* = 0.0195) and overall mortality (HR = 0.8, *p* = 0.0076) compared with patients < 50 years, an association not observed in the non-cc msRCC cohort. Racial disparities remained prominent in cc msRCC, with Black patients experiencing a significantly higher estimated hazard of cancer-specific mortality (HR = 1.4, *p* = 0.0008) and overall mortality (HR = 1.3, *p =* 0.0038); Hispanic patients also demonstrated worse survival outcomes (cancer-specific mortality and overall mortality HRs = 1.2; and *p* = 0.0115 and 0.0053, respectively) relative to White patients. The racial disparity persisted between Black and White patients within the non-cc msRCC cohort, with Black patients exhibiting worse cancer-specific mortality (HR = 1.8, *p* = 0.0051) and overall mortality (HR = 1.7, *p* = 0.0073).

Advanced tumor stage (T4) and lymph node involvement (N1) were independently associated with worse survival in both cc msRCC and non-cc msRCC. Age ≥ 80 years consistently predicted poor outcomes across histologic subtypes. Surgical intervention was strongly associated with improved survival in both cc msRCC (cancer-specific mortality and overall mortality HRs = 0.4, *p* < 0.0001) and non-cc msRCC (cancer-specific mortality and overall mortality HRs = 0.5, *p* = 0.0002 and 0.0007, respectively). Systemic therapy was also associated with significantly improved survival in both subtypes, with a greater magnitude of benefit observed in non-cc msRCC (both CSS and OS HRs = 0.6, *p* = 0.0004 and 0.0005, respectively). Finally, diagnosis during the ICI era (2016–2020) was associated with significantly improved survival in cc subtypes (HRs = 0.6, *p* < 0.0001 for both cancer-specific mortality and overall mortality) in cc msRCC, and marginally improved survival (HR = 0.8, *p* = 0.0685 for CSS, and HR = 0.8, *p =* 0.0508 for OS) underscoring the substantial impact of immunotherapy on msRCC outcomes.

## 4. Discussion

Our study provides the largest retrospective analysis of racial disparities in msRCC to date, revealing significantly worse survival outcomes for Black patients compared to other racial groups. While previous studies have documented racial disparities in RCC incidence and survival [7,8,9,10], few have specifically examined msRCC, an aggressive subtype with a historically poor prognosis [1,2].

### 4.1. Black Patients Exhibited the Worst Survival Among All Racial Groups

Our findings showed that Black patients had the worst observed 3-year CSS and OS among patients with msRCC, while other racial minority groups did not exhibit statistically significant survival differences compared to White patients. Our multivariable analyses suggest that several factors may have been associated with the survival difference. First, Black patients were the least likely to undergo surgery, with only 61.0% receiving surgical intervention. Surgery was the strongest factor associated with improved survival in the cohort, conferring a 60% lower estimated hazard of both cancer-specific mortalityCSS and overalll mortality (HRs = 0.4, *p* < 0.0001). Prior studies have suggested differences in nephrectomy rates between the 2 groups in mRCC [8]. An analysis of 31,989 mRCC patients from the National Cancer Database (2004–2015) found that Black patients had significantly increased mortality (HR = 1.06, *p* = 0.03) compared to White patients and were significantly less likely to receive cytoreductive nephrectomy (OR 0.75). Of note, their survival difference was eliminated after adjusting for treatment receipt (HR 0.99, *p* = 0.66) [14]. In an analysis of nearly 40,000 patients with RCC from the SEER database (1992–2007) [15], Black patients had a lower 5-year relative survival rate (68.0%) compared to White patients (72.6%) and were more likely to receive no surgical treatment (14.5% of Black patients versus 10.5% of White patients). Interestingly, there was no significant difference in survival between Black and White patients within the nephrectomy group. These findings suggest that access to surgery may play a key role in equalizing outcomes for these racial groups.

Radiotherapy was associated with worse survival in our multivariable analysis; however, this finding should be interpreted cautiously. In patients with metastatic RCC, radiotherapy is frequently used for palliation of symptomatic or high-burden metastatic disease, such as bone or brain metastases. Therefore, receipt of radiotherapy likely represents a marker of more advanced or symptomatic disease rather than an independent cause of poorer outcomes. Because SEER lacks detailed information on metastatic burden, treatment intent, and disease progression, residual confounding by indication cannot be excluded. Accordingly, this association should not be interpreted as evidence that radiotherapy causes poorer survival.

Socioeconomic factors may also have been associated with the observed disparities. Black patients had the highest rates of living in low-income areas and unmarried status. Our data suggest that patients with area-level incomes ≥ $75,000 experienced significantly improved survival outcomes. Prior studies in RCC have also shown that low-income areas and unmarried statuses may have been associated with Black patients’ poor survival outcomes [16,17,18,19].

Although Black patients have been reported to present at a younger age with lower-stage disease, they consistently experience worse OS [7]. This suggests that disparities in sociodemographic factors, treatment access, and underlying biological differences may be associated with differences in outcomes. It is important to note that, even after adjustment for other measured factors, Black patients continued to experience significantly worse CSS and OS. These disparities may reflect the cumulative effects of structural inequities, differences in access to and quality of care, treatment patterns, comorbidity burden, and other unmeasured factors, while potential biological mechanisms require further investigation.

Black patients also presented with the highest proportion of non-cc msRCC (20.7%). This finding aligns with previous studies showing Black patients have a higher proportion of non-cc RCC [15], specifically the papillary subtype [20,21,22]. A retrospective analysis suggests that this propensity for the papillary subtype is likely associated with a higher prevalence of comorbidities such as hypertension, diabetes, and chronic kidney disease within the Black population [23].

Interestingly, a retrospective study of mRCC patients enrolled in clinical trials at Wayne State University from 1992–2002 showed that even in the clinical trial setting where social effects were reduced, Black patients had significantly shorter OS; multivariate analysis identified race as a significant independent predictor of shorter survival than White patients (*p* = 0.0027) [24]. This suggests the potential need to look for biomarkers that could contribute to a worse prognosis of Black patients with mRCC. Investigating biological factors underlying differential treatment responses can inform precision medicine approaches. A case–control analysis using The Cancer Genome Atlas (TCGA) and the Chinese OrigiMed2020 cohort showed a higher prevalence of MiT family translocation in Asian and Black patients with RCC compared with White patients and showed that these tumors in Asian and Black patients have distinct transcriptional signatures [25]. An analysis performed with TCGA data identified 21 genes differentially expressed between Black and White patients that significantly affected survival [26]. Interestingly, a recent study from the French Kidney Cancer Research Network did not find race as an independent prognostic factor for survival, suggesting that differences in healthcare systems may influence racial disparities [27].

It is important to interpret the observed disparities in the context of unmeasured social determinants and limitations inherent to the SEER database. Although our models adjusted for several sociodemographic characteristics, they did not capture their potential intersectional effects or factors such as education, insurance type, rural/urban residence, and neighborhood-level deprivation. SEER also lacks granular information on performance status, comorbidity burden, systemic treatment regimens, treatment adherence, referral patterns, and institutional expertise. These unmeasured social, clinical, and healthcare-system factors may contribute to residual differences in survival after adjustment. Notably, prior studies have reported attenuation or elimination of racial survival differences after accounting for treatment receipt, underscoring the potential role of healthcare access and delivery in shaping outcomes. Accordingly, the observed associations should not be interpreted as independent or causal effects of race. Potential biological mechanisms, including differences in tumor microenvironment, immune infiltration, genomic variation, and pharmacogenomic factors, remain hypothesis-generating and require prospective validation, particularly given their complex interplay with socioeconomic conditions, healthcare access, treatment receipt, and clinical trial participation.

### 4.2. Treatment Advances Have Not Benefited All Racial Groups Equally

Despite notable survival improvements in most racial groups after the introduction of ICIs, racial disparities persisted. Our study found that post-ICI introduction, Hispanic and Asian and Pacific Islander patients saw the greatest survival gains, achieving survival outcomes that were no longer significantly different from those of White patients. However, Black patients remained the only group with persistently worse CSS and OS, with an 80% higher estimated hazard of cancer-specific mortality (HR = 1.8, *p* = 0.0002) and a 60% higher estimated hazard of overall mortality (HR = 1.6, *p* = 0.0012) compared to White patients. A recent analysis of SEER data from 2000–2020 for patients aged 15–39 years also showed that survival among Black patients remained poorer than other racial groups [3]. A retrospective study of 48,846 patients with stage IV RCC found that although survival improved for both groups after the advent of targeted therapy, the HR (1.13) for death for African Americans compared to White patients remained the same [28]. Gupta et al. analyzed 4858 mRCC patients receiving ICI therapy and found that Black patients had significantly shorter median OS (mOS: 14.8 months) compared to White (23.2 months) and Asian patients (22.2 months), even after adjusting for demographics and treatment factors [29]. Similarly, Olsen et al. examined 198 patients with mRCC treated with ICIs at a single academic center and found that Black patients had significantly shorter progression-free survival compared to White patients [30]. A cohort study of 8534 patients with metastatic ccRCC showed that although ICI-based combinations are the preferred first-line therapy, many patients were still receiving tyrosine kinase inhibitors (TKIs) or ICI monotherapy, suggesting a need to optimize treatment access and guideline adherence [31].

CheckMate 214 demonstrated improved overall survival (HR, 0.45), progression-free survival (HR, 0.54), and objective response (60.8% vs. 23.1%) with nivolumab plus ipilimumab versus sunitinib [32]. IMmotion151 and JAVELIN Renal 101 similarly showed improved progression-free survival and response outcomes with ICI-based combinations [33,34]. In contrast, Black patients in our SEER cohort continued to experience substantially poorer survival, suggesting a potential gap between clinical-trial efficacy and equitable real-world effectiveness. We now emphasize the need for implementation-science and equity-focused research. However, because SEER does not identify individual ICI use or specific regimens, our study cannot determine whether these disparities reflect differences in treatment access or outcomes among patients receiving the same therapies.

Furthermore, differences in immune profiles may contribute to differences in ICI response. An analysis of TCGA gene expression data suggested enrichment of 62 gene sets related to immune system pathways in Black patients compared with White patients, implicating a distinct immune response [35]. Several studies have implicated that differences in baseline immune and inflammatory markers could contribute to differences in treatment response to ICIs [36,37,38], suggesting the need for future studies to explore these biological differences more deeply to guide personalized treatment strategies and reduce outcome disparities.

It is important to note that a limitation of our study is that the SEER database records only whether systemic therapy was administered and does not specify the treatment regimen. Therefore, although survival improved during the era when ICIs became widely adopted, these gains cannot be definitively attributed to ICI use, nor can we determine whether the persistent racial disparities reflect differences in access to contemporary ICI-based therapies or differences in outcomes among patients receiving the same standard-of-care regimens.

### 4.3. Racial Disparities Between Black and White Patients Persisted in the Clear Cell msRCC and Non-Clear Cell msRCC Subtypes

Our study found that in both cc msRCC and non-cc msRCC subtypes, Black patients experienced significantly worse observed 3-year CSS and OS rates than White patients. Furthermore, both univariable and multivariable Cox regression analyses demonstrated that Black patients had significantly higher estimated hazards of cancer-specific and all-cause mortality compared with White patients, indicating persistent racial disparities in survival regardless of histologic subtype. An analysis of SEER data found that race was not associated with 5-year cause-specific survival in ccRCC, suggesting that differences in ccRCC survival may be driven by social determinants of health rather than cancer-specific factors [39]. Interestingly, previous studies have also shown that Black patients experienced higher mortality in ccRCC and lower mortality in non-ccRCC compared to White patients [40,41]. This underscores the complex interplay between race and ethnicity, tumor histology, and access to care, highlighting the need for future studies to understand histologic differences between racial groups and targeted efforts to address social determinants of health.

### 4.4. Study Limitations

This retrospective study has several limitations. First, its observational design is susceptible to selection bias, unmeasured confounding, and coding errors; therefore, causal relationships cannot be established. Sarcomatoid differentiation was identified using a SEER variable that does not provide information on the proportion or extent of the sarcomatoid component. Sample sizes were particularly limited for American Indian/Alaska Native patients, patients with unknown race or ethnicity, and Black patients with non–cc msRCC. These small subgroup sizes may reduce statistical power, increase the risk of Type II error, and yield unstable estimates with wide confidence intervals. Accordingly, findings for these subgroups should be interpreted cautiously and validated in larger cohorts. Treatment variables represented therapies recorded by SEER as part of the first course of treatment, whereas survival time was measured from diagnosis. Because SEER does not provide sufficiently detailed treatment dates to establish the exact timing of therapy during follow-up, treatment was modeled as a baseline covariate, potentially introducing immortal-time bias. SEER provides limited treatment information and does not capture specific systemic regimens, therapeutic agents, treatment sequences, dosages, treatment duration, responses, or subsequent therapies. In particular, the database does not identify patients who received ICIs or permit comparisons of ICI use across racial and ethnic groups. Therefore, although survival improved during the period when ICIs became widely adopted, this improvement cannot be definitively attributed to ICI use. Similarly, we could not determine whether persistent racial and ethnic disparities reflected differences in access to contemporary ICI-based therapies or differences in outcomes among patients receiving comparable standard-of-care regimens. Information on performance status (ECOG), comorbidities, laboratory findings, lifestyle factors, and other clinically relevant characteristics was unavailable. Consequently, the poorer survival observed among patients receiving radiotherapy may partly reflect confounding by indication, because these patients may have had more advanced disease or poorer baseline health. SEER also does not capture cancer recurrence or disease progression, precluding evaluation of disease-free or progression-free survival. Important factors, such as education level, health insurance type (e.g., Medicaid vs private), and rural/urban residence are not included. Finally, limited information on health insurance, family history, tumor molecular characteristics, and other socioeconomic and biological factors restricted our ability to investigate the mechanisms underlying racial and ethnic disparities in survival.

## 5. Conclusions

In summary, to our knowledge, in the largest retrospective analysis to date of msRCC using SEER data, we identified persistent and significant racial disparities in survival outcomes, particularly among Black patients. Despite advances in therapy—including the widespread adoption of ICIs—Black patients consistently had the worst CSS and OS across all histologic subtypes and time periods. These disparities appear to be multifactorial, stemming from differences in treatment access (notably lower surgical rates), socioeconomic disadvantage, and potential biological variations, and are evident in both clear cell and non-clear cell types. While most racial groups showed improved outcomes in the modern treatment era, Black patients remain at disproportionately high risk, underscoring a critical need for interventions that address systemic barriers, increase access to evidence-based treatments, and expand research into biological factors (such as microbiome, transcriptomic, immune-cell, or other molecular measurements) potentially associated with treatment resistance. However, it is important to note that race should be interpreted as a proxy for broader social and structural inequities, as the SEER database lacks detailed measures of healthcare access, insurance status, neighborhood deprivation, and quality of care that may contribute to these disparities. These findings should therefore be interpreted with caution and validated in larger, more diverse cohorts. Future studies must prioritize inclusive clinical trial designs, integrate biomarkers of response, and implement community-level strategies to ensure equitable care and improved survival for all patients with msRCC. More broadly, improving outcomes in msRCC will require not only continued advances in precision oncology but also equitable access to evidence-based diagnosis, specialized cancer care, and effective treatments. Addressing disparities will therefore require an integrated approach that considers both potential biological mechanisms and the social and healthcare-system factors that shape cancer outcomes. Ensuring that therapeutic advances translate equitably across populations should remain a central priority in improving msRCC care.

## Figures and Tables

**Table 1:** Baseline characteristics of patients with metastatic sarcomatoid renal cell cancer diagnosed during 2010–2020.

Variable	Overall *n* (%)	Hispanic	Non-Hispanic American Indian and Alaska Native	Non-Hispanic Asian and Pacific Islanders	Non-Hispanic Black	Non-Hispanic White	*p* Value
	**2122 (100.0)**	**365 (17.2)**	**30 (1.4)**	**117 (5.5)**	**169 (8.0)**	**1441 (67.9)**	
**Age (years)**							
**Groups**							<0.0001
**<50**	249 (11.7)	67 (18.4)	5 (16.7)	13 (11.1)	29 (17.2)	135 (9.4)	
**50–59**	600 (28.3)	117 (32.1)	11 (36.7)	40 (34.2)	51 (30.2)	381 (26.4)	
**60–69**	728 (34.3)	109 (29.9)	9 (30.0)	40 (34.2)	57 (33.7)	513 (35.6)	
**70–79**	420 (19.8)	59 (16.2)	5 (16.7)	19 (16.2)	20 (11.8)	317 (22.0)	
**≥80**	125 (5.9)	13 (3.6)	(0.0)	5 (4.3)	12 (7.1)	95 (6.6)	
**Sex**							0.0121
**Male**	1505 (70.9)	242 (66.3)	19 (63.3)	73 (62.4)	116 (68.6)	1055 (73.2)	
**Female**	617 (29.1)	123 (33.7)	11 (36.7)	44 (37.6)	53 (31.4)	386 (26.8)	
**Marital status**							< 0.0001
**Married**	1345 (63.4)	221 (60.6)	14 (46.7)	79 (67.5)	77 (45.6)	954 (66.2)	
**Unmarried**	716 (33.7)	138 (37.8)	13 (43.3)	33 (28.2)	87 (51.5)	445 (30.9)	
**Unknown**	61 (2.9)	6 (1.6)	3 (10.0)	5 (4.3)	5 (3.0)	42 (2.9)	
**Region**							< 0.0001
**West**	1132 (53.4)	311 (85.2)	22 (73.3)	104 (88.9)	55 (32.5)	640 (44.4)	
**South**	588 (27.7)	16 (4.4)	3 (10.0)	3 (2.6)	88 (52.1)	478 (33.2)	
**Midwest**	110 (5.2)	5 (1.4)	1 (3.3)	(0.0)	(0.0)	104 (7.2)	
**Northeast**	292 (13.8)	33 (9.0)	4 (13.3)	10 (8.6)	26 (15.4)	219 (15.2)	
**Income ($)**							< 0.0001
**Less than 60,000**	586 (27.6)	67 (18.4)	9 (30.0)	8 (6.8)	56 (33.1)	446 (31.0)	
**60,000–74,999**	769 (36.2)	165 (45.2)	7 (23.3)	40 (34.2)	65 (38.5)	492 (34.1)	
**75,000 and over**	767 (36.2)	133 (36.4)	14 (46.7)	69 (59.0)	48 (28.4)	503 (34.9)	
**T stage**							0.0025
**T1**	217 (10.2)	28 (7.7)	5 (16.7)	11 (9.4)	29 (17.2)	144 (10.0)	
**T2**	241 (11.4)	47 (12.9)	1 (3.3)	15 (12.8)	27 (16.0)	151 (10.5)	
**T3**	1102 (51.9)	173 (47.4)	16 (53.3)	62 (53.0)	72 (42.6)	779 (54.1)	
**T4**	410 (19.3)	94 (25.8)	5 (16.7)	21 (18.0)	25 (14.8)	265 (18.4)	
**Unknown**	152 (7.2)	23 (6.3)	3 (10.0)	8 (6.8)	16 (9.5)	102 (7.1)	
**N stage**							0.3641
**N0**	1190 (56.1)	211 (57.8)	17 (56.7)	70 (59.8)	89 (52.7)	803 (55.7)	
**N1**	770 (36.3)	127 (34.8)	8 (26.7)	35 (29.9)	65 (38.5)	535 (37.1)	
**Unknown**	162 (7.6)	27 (7.4)	5 (16.7)	12 (10.3)	15 (8.9)	103 (7.2)	
**Tumor size (cm)**							0.3282
**≤3**	68 (3.2)	8 (2.2)	(0.0)	7 (6.0)	8 (4.7)	45 (3.1)	
**>3**	1938 (91.3)	340 (93.2)	30 (100.0)	104 (88.9)	152 (89.9)	1312 (91.1)	
**Unknown**	116 (5.5)	17 (4.7)	(0.0)	6 (5.1)	9 (5.3)	84 (5.8)	
**Histology**							0.0042
**Clear cell**	1740 (82.0)	310 (84.9)	28 (93.3)	96 (82.1)	134 (79.3)	1172 (81.3)	
**Papillary**	57 (2.7)	11 (3.0)	(0.0)	5 (4.3)	13 (7.7)	28 (1.9)	
**Chromophobe**	25 (1.2)	4 (1.1)	(0.0)	1 (0.9)	2 (1.2)	18 (1.3)	
**Other**	300 (14.1)	40 (11.0)	2 (6.7)	15 (12.8)	20 (11.8)	223 (15.5)	
**Grade**							0.1428
**I**	14 (0.7)	3 (0.8)	1 (3.3)	1 (0.9)	1 (0.6)	8 (0.6)	
**II**	40 (1.9)	12 (3.3)	1 (3.3)	3 (2.6)	3 (1.8)	21 (1.5)	
**III/IV**	1141 (53.8)	185 (50.7)	18 (60.0)	68 (58.1)	78 (46.2)	792 (55.0)	
**Unknown**	927 (43.7)	165 (45.2)	10 (33.3)	45 (38.5)	87 (51.5)	620 (43.0)	
**Surgery**							0.0293
**No**	609 (28.7)	106 (29.0)	8 (26.7)	28 (23.9)	66 (39.1)	401 (27.8)	
**Yes**	1513 (71.3)	259 (71.0)	22 (73.3)	89 (76.1)	103 (61.0)	1040 (72.2)	
**Systemic Therapy**							0.7514
**No**	1064 (50.1)	193 (52.9)	13 (43.3)	59 (50.4)	82 (48.5)	717 (49.8)	
**Yes**	1058 (49.9)	172 (47.1)	17 (56.7)	58 (49.6)	87 (51.5)	724 (50.2)	
**Radiotherapy**							0.4818
**No**	1531 (72.2)	267 (73.2)	22 (73.3)	86 (73.5)	131 (77.5)	1025 (71.1)	
**Yes**	591 (27.9)	98 (26.9)	8 (26.7)	31 (26.5)	38 (22.5)	416 (28.9)	
**Cancer-specific death**							0.0507
**No**	595 (28.0)	111 (30.4)	10 (33.3)	34 (29.1)	31 (18.3)	409 (28.4)	
**Yes**	1527 (72.0)	254 (69.6)	20 (66.7)	83 (70.9)	138 (81.7)	1032 (71.6)	
**Overall death**							0.1939
**No**	466 (22.0)	85 (23.3)	7 (23.3)	29 (24.8)	25 (14.8)	320 (22.2)	
**Yes**	1656 (78.0)	280 (76.7)	23 (76.7)	88 (75.2)	144 (85.2)	1121 (77.8)	
**Diagnosis year**							0.0843
**2010–2015**	1080 (50.9)	163 (44.7)	15 (50.0)	67 (57.3)	87 (51.5)	748 (51.9)	
**2016–2020**	1042 (49.1)	202 (55.3)	15 (50.0)	50 (42.7)	82 (48.5)	693 (48.1)	

**Table 2:** Multivariable survival analysis of factors associated with cancer-specific and overall survival in patients with metastatic sarcomatoid RCC diagnosed during 2010–2020.

Variable	Cancer-Specific Survival		Overall Survival	
	HR (95% CI)	*p* Value	HR (95% CI)	*p* Value
**Histology**				
**Clear cell**	1		1	
**Papillary**	0.9 (0.6–1.2)	0.3891	0.9 (0.7–1.2)	0.3876
**Chromophobe**	1.8 (1.1–2.8)	0.0167	1.8 (1.2–2.8)	0.008
**Other**	1.2 (1.1–1.4)	0.0064	1.2 (1.03–1.4)	0.018
**Diagnosis year**				
**2010–2015**	1		1	
**2016–2020**	0.6 (0.5–0.7)	<0.0001	0.6 (0.6–0.7)	<0.0001
**Age (years)**				
**Groups**				
**<50**	1		1	
**50–59**	0.9 (0.7–1.02)	0.078	0.8 (0.7–0.98)	0.0282
**60–69**	1.0 (0.8–1.2)	0.8372	1.0 (0.8–1.2)	0.9122
**70–79**	1.1 (0.9–1.3)	0.3607	1.1 (0.9–1.3)	0.3636
**≥80**	1.7 (1.3–2.2)	<0.0001	1.9 (1.5–2.4)	<0.0001
**Race**				
**Hispanic**	1.1 (0.99–1.3)	0.0663	1.2 (1.02–1.3)	0.0249
**Non-Hispanic American Indian and Alaska Native**	0.9 (0.6–1.4)	0.686	1.0 (0.7–1.5)	0.9435
**Non-Hispanic Asian and Pacific Islander**	1.1 (0.9–1.4)	0.2978	1.1 (0.9–1.4)	0.349
**Non-Hispanic Black**	1.5 (1.3–1.8)	<0.0001	1.5 (1.2–1.7)	<0.0001
**Non-Hispanic White**	1		1	
**Income ($)**				
**Less than 60,000**	1		1	
**60,000–74,999**	1.0 (0.9–1.1)	0.6455	1.0 (0.9–1.1)	0.8436
**75,000 and over**	0.9 (0.8–0.97)	0.0177	0.8 (0.7–0.95)	0.0068
**T stage**				
**T1**	1		1	
**T2**	1.1 (0.9–1.4)	0.2382	1.2 (0.9–1.4)	0.1736
**T3**	1.1 (0.9–1.4)	0.1646	1.1 (0.9–1.4)	0.1661
**T4**	1.7 (1.4–2.0)	<0.0001	1.7 (1.4–2.1)	<0.0001
**Unknown**	1.4 (1.04–1.8)	0.0254	1.4 (1.1–1.8)	0.0056
**N stage**				
**N0**	1		1	
**N1**	1.5 (1.4–1.7)	<0.0001	1.5 (1.4–1.7)	<0.0001
**Unknown**	1.1 (0.9–1.4)	0.2282	1.2 (1.0–1.4)	0.1352
**Surgery**				
**No**	1		1	
**Yes**	0.4 (0.3–0.5)	<0.0001	0.4 (0.3–0.4)	<0.0001
**Systemic Therapy**				
**No**	1		1	
**Yes**	0.7 (0.6–0.8)	<0.0001	0.7 (0.6–0.7)	<0.0001
**Radiotherapy**				
**No**	1		1	
**Yes**	1.2 (1.0–1.3)	0.0109	1.1 (1.01–1.3)	0.0349

**Table 3:** Baseline characteristics of patients with metastatic sarcomatoid renal cell cancer diagnosed during 2010–2015.

Variable	Overall *n* (%)	Hispanic	Non-Hispanic American Indian and Alaska Native	Non-Hispanic Asian and Pacific Islander	Non-Hispanic Black	Non-Hispanic White	*p* Value
	**1080 (100.0)**	**163 (15.1)**	**15 (1.4)**	**67 (6.2)**	**87 (8.1)**	**748 (69.3)**	
**Age (years)**							
**Groups**							0.0032
**< 50**	120 (11.1)	32 (19.6)	3 (20.0)	8 (11.9)	14 (16.1)	63 (8.4)	
**50–59**	332 (30.7)	55 (33.7)	7 (46.7)	24 (35.8)	25 (28.7)	221 (29.6)	
**60–69**	372 (34.4)	42 (25.8)	2 (13.3)	20 (29.9)	34 (39.1)	274 (36.6)	
**70–79**	196 (18.2)	29 (17.8)	3 (20.0)	12 (17.9)	9 (10.3)	143 (19.1)	
**≥ 80**	60 (5.6)	5 (3.1)	(0.0)	3 (4.5)	5 (5.8)	47 (6.3)	
**Sex**							0.0126
**Male**	769 (71.2)	105 (64.4)	13 (86.7)	39 (58.2)	63 (72.4)	549 (73.4)	
**Female**	311 (28.8)	58 (35.6)	2 (13.3)	28 (41.8)	24 (27.6)	199 (26.6)	
**Marital status**							0.2498
**Married**	683 (63.2)	104 (63.8)	8 (53.3)	40 (59.7)	48 (55.2)	483 (64.6)	
**Unmarried**	366 (33.9)	57 (35.0)	6 (40.0)	23 (34.3)	38 (43.7)	242 (32.4)	
**Unknown**	31 (2.9)	2 (1.2)	1 (6.7)	4 (6.0)	1 (1.2)	23 (3.1)	
**Region**							< 0.0001
**West**	585 (54.2)	139 (85.3)	11 (73.3)	62 (92.5)	31 (35.6)	342 (45.7)	
**South**	300 (27.8)	8 (4.9)	1 (6.7)	2 (3.0)	41 (47.1)	248 (33.2)	
**Midwest**	56 (5.2)	1 (0.6)	1 (6.7)	(0.0)	(0.0)	54 (7.2)	
**Northeast**	139 (12.9)	15 (9.2)	2 (13.3)	3 (4.5)	15 (17.2)	104 (13.9)	
**Income ($)**							< 0.0001
**Less than 60,000**	338 (31.3)	35 (21.5)	6 (40.0)	3 (4.5)	28 (32.2)	266 (35.6)	
**60,000–74,999**	422 (39.1)	89 (54.6)	4 (26.7)	28 (41.8)	36 (41.4)	265 (35.4)	
**75,000 and over**	320 (29.6)	39 (23.9)	5 (33.3)	36 (53.7)	23 (26.4)	217 (29.0)	
**T stage**							0.0104
**T1**	109 (10.1)	10 (6.1)	1 (6.7)	6 (9.0)	13 (14.9)	79 (10.6)	
**T2**	109 (10.1)	18 (11.0)	(0.0)	5 (7.5)	18 (20.7)	68 (9.1)	
**T3**	581 (53.8)	87 (53.4)	11 (73.3)	36 (53.7)	35 (40.2)	412 (55.1)	
**T4**	217 (20.1)	42 (25.8)	1 (6.7)	15 (22.4)	13 (14.9)	146 (19.5)	
**Unknown**	64 (5.9)	6 (3.7)	2 (13.3)	5 (7.5)	8 (9.2)	43 (5.8)	
**N stage**							0.0248
**N0**	602 (55.7)	92 (56.4)	10 (66.7)	39 (58.2)	40 (46.0)	421 (56.3)	
**N1**	410 (38.0)	60 (36.8)	2 (13.3)	20 (29.9)	39 (44.8)	289 (38.6)	
**Unknown**	68 (6.3)	11 (6.8)	3 (20.0)	8 (11.9)	8 (9.2)	38 (5.1)	
**Tumor size (cm)**							0.5714
**≤3**	42 (3.9)	5 (3.1)	(0.0)	5 (7.5)	4 (4.6)	28 (3.7)	
**>3**	991 (91.8)	151 (92.6)	15 (100.0)	57 (85.1)	81 (93.1)	687 (91.8)	
**Unknown**	47 (4.4)	7 (4.3)	(0.0)	5 (7.5)	2 (2.3)	33 (4.4)	
**Histology**							0.0096
**Clear cell**	825 (76.4)	129 (79.1)	14 (93.3)	50 (74.6)	64 (73.6)	568 (75.9)	
**Papillary**	27 (2.5)	5 (3.1)	(0.0)	4 (6.0)	7 (8.1)	11 (1.5)	
**Chromophobe**	10 (0.9)	1 (0.6)	(0.0)	1 (1.5)	(0.0)	8 (1.1)	
**Other**	218 (20.2)	28 (17.2)	1 (6.7)	12 (17.9)	16 (18.4)	161 (21.5)	
**Grade**							0.1661
**I**	3 (0.3)	(0.0)	(0.0)	(0.0)	(0.0)	3 (0.4)	
**II**	19 (1.8)	5 (3.1)	(0.0)	(0.0)	(0.0)	14 (1.9)	
**III/IV**	761 (70.5)	116 (71.2)	13 (86.7)	46 (68.7)	51 (58.6)	535 (71.5)	
**Unknown**	297 (27.5)	42 (25.8)	2 (13.3)	21 (31.3)	36 (41.4)	196 (26.2)	
**Surgery**							0.0085
**No**	275 (25.5)	41 (25.2)	2 (13.3)	16 (23.9)	36 (41.4)	180 (24.1)	
**Yes**	805 (74.5)	122 (74.9)	13 (86.7)	51 (76.1)	51 (58.6)	568 (75.9)	
**Systemic Therapy**							0.9134
**No**	460 (42.6)	74 (45.4)	6 (40.0)	30 (44.8)	38 (43.7)	312 (41.7)	
**Yes**	620 (57.4)	89 (54.6)	9 (60.0)	37 (55.2)	49 (56.3)	436 (58.3)	
**Radiotherapy**							0.1299
**No**	765 (70.8)	108 (66.3)	13 (86.7)	50 (74.6)	69 (79.3)	525 (70.2)	
**Yes**	315 (29.2)	55 (33.7)	2 (13.3)	17 (25.4)	18 (20.7)	223 (29.8)	
**Cancer-specific death**							0.1274
**No**	151 (14.0)	24 (14.7)	4 (26.7)	10 (14.9)	5 (5.8)	108 (14.4)	
**Yes**	929 (86.0)	139 (85.3)	11 (73.3)	57 (85.1)	82 (94.3)	640 (85.6)	
**Overall death**							0.2142
**No**	90 (8.3)	14 (8.6)	3 (20.0)	7 (10.5)	3 (3.5)	63 (8.4)	
**Yes**	990 (91.7)	149 (91.4)	12 (80.0)	60 (89.6)	84 (96.6)	685 (91.6)	

**Table 4:** Baseline characteristics of patients with metastatic sarcomatoid renal cell cancer diagnosed during 2016–2020.

Variable	Overall *n* (%)	Hispanic	Non-Hispanic American Indian and Alaska Native	Non-Hispanic Asian and Pacific Islander	Non-Hispanic Black	Non-Hispanic White	*p* Value
	**1042 (100.0)**	**202 (19.4)**	**15 (1.4)**	**50 (4.8)**	**82 (7.9)**	**693 (66.5)**	
**Age (years)**							
**Groups**							0.0063
**<50**	129 (12.4)	35 (17.3)	2 (13.3)	5 (10.0)	15 (18.3)	72 (10.4)	
**50–59**	268 (25.7)	62 (30.7)	4 (26.7)	16 (32.0)	26 (31.7)	160 (23.1)	
**60–69**	356 (34.2)	67 (33.2)	7 (46.7)	20 (40.0)	23 (28.1)	239 (34.5)	
**70–79**	224 (21.5)	30 (14.9)	2 (13.3)	7 (14.0)	11 (13.4)	174 (25.1)	
**≥80**	65 (6.2)	8 (4.0)	(0.0)	2 (4.0)	7 (8.5)	48 (6.9)	
**Sex**							0.0261
**Male**	736 (70.6)	137 (67.8)	6 (40.0)	34 (68.0)	53 (64.6)	506 (73.0)	
**Female**	306 (29.4)	65 (32.2)	9 (60.0)	16 (32.0)	29 (35.4)	187 (27.0)	
**Marital status**							<0.0001
**Married**	662 (63.5)	117 (57.9)	6 (40.0)	39 (78.0)	29 (35.4)	471 (68.0)	
**Unmarried**	350 (33.6)	81 (40.1)	7 (46.7)	10 (20.0)	49 (59.8)	203 (29.3)	
**Unknown**	30 (2.9)	4 (2.0)	2 (13.3)	1 (2.0)	4 (4.9)	19 (2.7)	
**Region**							<0.0001
**West**	547 (52.5)	172 (85.2)	11 (73.3)	42 (84.0)	24 (29.3)	298 (43.0)	
**South**	288 (27.6)	8 (4.0)	2 (13.3)	1 (2.0)	47 (57.3)	230 (33.2)	
**Midwest**	54 (5.2)	4 (2.0)	(0.0)	(0.0)	(0.0)	50 (7.2)	
**Northeast**	153 (14.7)	18 (8.9)	2 (13.3)	7 (14.0)	11 (13.4)	115 (16.6)	
**Income ($)**							0.0003
**Less than 60,000**	248 (23.8)	32 (15.8)	3 (20.0)	5 (10.0)	28 (34.2)	180 (26.0)	
**60,000–74,999**	347 (33.3)	76 (37.6)	3 (20.0)	12 (24.0)	29 (35.4)	227 (32.8)	
**75,000 and over**	447 (42.9)	94 (46.5)	9 (60.0)	33 (66.0)	25 (30.5)	286 (41.3)	
**T stage**							0.0240
**T1**	108 (10.4)	18 (8.9)	4 (26.7)	5 (10.0)	16 (19.5)	65 (9.4)	
**T2**	132 (12.7)	29 (14.4)	1 (6.7)	10 (20.0)	9 (11.0)	83 (12.0)	
**T3**	521 (50.0)	86 (42.6)	5 (33.3)	26 (52.0)	37 (45.1)	367 (53.0)	
**T4**	193 (18.5)	52 (25.7)	4 (26.7)	6 (12.0)	12 (14.6)	119 (17.2)	
**Unknown**	88 (8.5)	17 (8.4)	1 (6.7)	3 (6.0)	8 (9.8)	59 (8.5)	
**N stage**							0.9464
**N0**	588 (56.4)	119 (58.9)	7 (46.7)	31 (62.0)	49 (59.8)	382 (55.1)	
**N1**	360 (34.6)	67 (33.2)	6 (40.0)	15 (30.0)	26 (31.7)	246 (35.5)	
**Unknown**	94 (9.0)	16 (7.9)	2 (13.3)	4 (8.0)	7 (8.5)	65 (9.4)	
**Tumor size (cm)**							0.3807
**≤3**	26 (2.5)	3 (1.5)	(0.0)	2 (4.0)	4 (4.9)	17 (2.5)	
**>3**	947 (90.9)	189 (93.6)	15 (100.0)	47 (94.0)	71 (86.6)	625 (90.2)	
**Unknown**	69 (6.6)	10 (5.0)	(0.0)	1 (2.0)	7 (8.5)	51 (7.4)	
**Histology**							0.6619
**Clear cell**	915 (87.8)	181 (89.6)	14 (93.3)	46 (92.0)	70 (85.4)	604 (87.2)	
**Papillary**	30 (2.9)	6 (3.0)	(0.0)	1 (2.0)	6 (7.3)	17 (2.5)	
**Chromophobe**	15 (1.4)	3 (1.5)	(0.0)	(0.0)	2 (2.4)	10 (1.4)	
**Other**	82 (7.9)	12 (5.9)	1 (6.7)	3 (6.0)	4 (4.9)	62 (9.0)	
**Grade**							0.0473
**I**	11 (1.1)	3 (1.5)	1 (6.7)	1 (2.0)	1 (1.2)	5 (0.7)	
**II**	21 (2.0)	7 (3.5)	1 (6.7)	3 (6.0)	3 (3.7)	7 (1.0)	
**III/IV**	380 (36.5)	69 (34.2)	5 (33.3)	22 (44.0)	27 (32.9)	257 (37.1)	
**Unknown**	630 (60.5)	123 (60.9)	8 (53.3)	24 (48.0)	51 (62.2)	424 (61.2)	
**Surgery**							0.6080
**No**	334 (32.1)	65 (32.2)	6 (40.0)	12 (24.0)	30 (36.6)	221 (31.9)	
**Yes**	708 (68.0)	137 (67.8)	9 (60.0)	38 (76.0)	52 (63.4)	472 (68.1)	
**Systemic Therapy**							0.8180
**No**	604 (58.0)	119 (58.9)	7 (46.7)	29 (58.0)	44 (53.7)	405 (58.4)	
**Yes**	438 (42.0)	83 (41.1)	8 (53.3)	21 (42.0)	38 (46.3)	288 (41.6)	
**Radiotherapy**							0.2755
**No**	766 (73.5)	159 (78.7)	9 (60.0)	36 (72.0)	62 (75.6)	500 (72.2)	
**Yes**	276 (26.5)	43 (21.3)	6 (40.0)	14 (28.0)	20 (24.4)	193 (27.9)	
**Cancer-specific death**							0.3050
**No**	444 (42.6)	87 (43.1)	6 (40.0)	24 (48.0)	26 (31.7)	301 (43.4)	
**Yes**	598 (57.4)	115 (56.9)	9 (60.0)	26 (52.0)	56 (68.3)	392 (56.6)	
**Overall death**							0.2525
**No**	376 (36.1)	71 (35.2)	4 (26.7)	22 (44.0)	22 (26.8)	257 (37.1)	
**Yes**	666 (63.9)	131 (64.9)	11 (73.3)	28 (56.0)	60 (73.2)	436 (62.9)	

**Table 5:** Multivariable survival analysis of factors associated with cancer-specific and overall survival in patients with metastatic sarcomatoid RCC diagnosed during 2010–2015 compared to 2016–2020.

Variable	2010–2015				2016–2020			
	Cancer-Specific Survival		Overall Survival		Cancer-Specific Survival		Overall Survival	
	HR (95% CI)	*p* Value	HR (95% CI)	*p* Value	HR (95% CI)	*p* Value	HR (95% CI)	*p* Value
**Histology**								
**Clear cell**	1		1		1		1	
**Papillary**	0.6 (0.4–0.9)	0.0256	0.6 (0.4–0.9)	0.0214	1.4 (0rac.9–2.2)	0.1248	1.4 (0.9–2.2)	0.1149
**Chromophobe**	1.4 (0.7–2.8)	0.3433	1.4 (0.7–2.8)	0.2755	2.0 (1.1–3.8)	0.0301	2.2 (1.2–3.9)	0.0131
**Other**	1.1 (0.96–1.3)	0.1483	1.1 (0.96–1.3)	0.1334	1.6 (1.2–2.1)	0.0009	1.4 (1.1–1.8)	0.0139
**Age (years)**								
**Groups**								
**<50**	1		1		1		1	
**50–59**	0.9 (0.7–1.1)	0.2856	0.9 (0.7–1.1)	0.1969	0.8 (0.6–1.03)	0.0822	0.7 (0.6–0.97)	0.0268
**60–69**	1.1 (0.9–1.3)	0.5823	1.1 (0.9–1.4)	0.4073	0.9 (0.7–1.1)	0.318	0.9 (0.7–1.1)	0.347
**70–79**	1.2 (0.9–1.5)	0.1703	1.2 (0.9–1.5)	0.2107	0.9 (0.7–1.2)	0.6112	0.9 (0.7–1.2)	0.5743
**≥80**	1.9 (1.3–2.6)	0.0003	2.0 (1.5–2.8)	<0.0001	1.6 (1.1–2.3)	0.0272	1.7 (1.2–2.4)	0.0042
**Race**								
**Hispanic**	1.1 (0.9–1.3)	0.5902	1.1 (0.9–1.3)	0.5134	1.1 (0.9–1.4)	0.3188	1.3 (1.03–1.5)	0.0247
**Non-Hispanic American Indian and Alaska Native**	0.8 (0.4–1.4)	0.4376	1.1 (0.8–1.4)	0.4804	1.0 (0.7–1.5)	0.945	1.1 (0.7–1.6)	0.7085
**Non-Hispanic Asian and Pacific Islander**	1.1 (0.8–1.5)	0.4222	0.8 (0.5–1.4)	0.4651	1.0 (0.5–1.9)	0.9251	1.2 (0.6–2.1)	0.6242
**Non-Hispanic Black**	1.5 (1.1–1.8)	0.0025	1.4 (1.1–1.7)	0.01	1.8 (1.3–2.4)	0.0002	1.6 (1.2–2.1)	0.0012
**Non-Hispanic White**	1		1		1		1	
**Region**								
**West**					1			
**South**					0.8 (0.7–0.998)	0.0476		
**Midwest**					1.1 (0.8–1.6)	0.603		
**Northeast**					0.8 (0.6–0.97)	0.0306		
**Income ($)**								
**Less than 60,000**	1		1					
**60,000–74,999**	0.9 (0.8–1.1)	0.3226	0.9 (0.8–1.1)	0.3316				
**75,000 and over**	0.8 (0.7–0.96)	0.0163	0.8 (0.7–0.95)	0.0111				
**T stage**								
**T1**	1		1		1		1	
**T2**	1.0 (0.8–1.4)	0.8532	1.0 (0.7–1.3)	0.9095	1.2 (0.9–1.7)	0.2206	1.3 (0.9–1.8)	0.1201
**T3**	1.2 (0.9–1.5)	0.2374	1.1 (0.9–1.4)	0.3225	1.1 (0.8–1.5)	0.5741	1.1 (0.8–1.5)	0.4408
**T4**	1.8 (1.3–2.3)	<0.0001	1.7 (1.4–2.2)	<0.0001	1.6 (1.1–2.2)	0.0064	1.6 (1.2–2.2)	0.0018
**Unknown**	1.3 (0.9–1.9)	0.1134	1.4 (0.994–2.0)	0.0538	1.4 (0.95–2.1)	0.086	1.4 (0.998–2.1)	0.0511
**N stage**								
**N0**	1		1		1		1	
**N1**	1.5 (1.3–1.8)	<0.0001	1.6 (1.4–1.8)	<0.0001	1.6 (1.3–1.9)	<0.0001	1.5 (1.3–1.8)	<0.0001
**Unknown**	1.2 (0.9–1.6)	0.2516	1.2 (0.9–1.6)	0.1953	1.1 (0.8–1.5)	0.6153	1.2 (0.9–1.6)	0.3014
**Surgery**								
**No**	1		1		1		1	
**Yes**	0.4 (0.3–0.5)	<0.0001	0.4 (0.3–0.5)	<0.0001	0.4 (0.3–0.4)	<0.0001	0.3 (0.3–0.4)	<0.0001
**Systemic Therapy**								
**No**	1		1		1		1	
**Yes**	0.6 (0.5–0.6)	<0.0001	0.5 (0.5–0.6)	<0.0001	0.9 (0.7–1.0)	0.0785	0.9 (0.7–1.02)	0.0453
**Radiotherapy**								
**No**	1							
**Yes**	1.2 (1.0–1.3)	0.0404						

Variables that were not significantly associated with survival outcomes (*p* ≥ 0.1) were excluded from the final models. Consequently, no estimates are reported for these variables in the table.

**Table 6:** Multivariable survival analysis of factors associated with cancer-specific and overall survival in patients with ccRCC vs. non-ccRCC subtypes with msRCC diagnosed during 2010–2020.

Variable	Clear Cell				Non-Clear Cell			
	Cancer-Specific Survival		Overall Survival		Cancer-Specific Survival		Overall Survival	
	HR (95% CI)	*p* Value	HR (95% CI)	*p* Value	HR (95% CI)	*p* Value	HR (95% CI)	*p* Value
**Age (years)**								
**Groups**								
**<50**	1		1		1		1	
**50–59**	0.8 (0.6–0.96)	0.0195	0.8 (0.6–0.9)	0.0076	1.2 (0.8–1.8)	0.3586	1.1 (0.8–1.6)	0.556
**60–69**	1.0 (0.8–1.2)	0.8194	1.0 (0.8–1.2)	0.8381	0.9 (0.6–1.4)	0.7396	0.9 (0.7–1.4)	0.7819
**70–79**	1.1 (0.9–1.3)	0.4836	1.1 (0.9–1.3)	0.5744	1.1 (0.7–1.7)	0.6531	1.1 (0.8–1.7)	0.5838
**≥80**	1.6 (1.2–2.2)	0.0018	1.8 (1.4–2.4)	<0.0001	2.3 (1.3–3.9)	0.0026	2.3 (1.4–3.9)	0.0017
**Race**								
**Hispanic**	1.2 (1.05–1.4)	0.0115	1.2 (1.1–1.4)	0.0053	0.8 (0.6–1.2)	0.2586	0.9 (0.6–1.2)	0.4328
**Non-Hispanic American Indian and Alaska Native**	1.1 (0.9–1.5)	0.357	1.1 (0.9–1.4)	0.3754	0.8 (0.5–1.4)	0.5019	0.9 (0.5–1.4)	0.5868
**Non-Hispanic Asian and Pacific Islander**	1.0 (0.6–1.6)	0.9766	1.1 (0.7–1.6)	0.8151	0.4 (0.1–2.9)	0.3574	0.7 (0.2–2.8)	0.58
**Non-Hispanic Black**	1.4 (1.2–1.8)	0.0008	1.3 (1.1–1.6)	0.0038	1.8 (1.2–2.7)	0.0051	1.7 (1.2–2.6)	0.0073
**Non-Hispanic White**	1		1		1		1	
**Region**								
**West**					0.7 (0.5–1.1)	0.1143	0.7 (0.5–1.1)	0.1519
**South**					0.6 (0.4–0.9)	0.0112	0.6 (0.4–0.95)	0.0288
**Midwest**					1		1	
**Northeast**					0.5 (0.3–0.8)	0.0093	0.6 (0.3–0.9)	0.0239
**Income ($)**								
**Less than 60,000**	1		1					
**60,000–74,999**	1.0 (0.9–1.1)	0.8899	1.0 (0.9–1.2)	0.8345				
**75,000 and over**	0.9 (0.8–1.01)	0.0655	0.9 (0.7–0.98)	0.0273				
**T stage**								
**T1**	1		1		1		1	
**T2**	1.1 (0.9–1.4)	0.3534	1.1 (0.9–1.4)	0.4356	1.2 (0.7–2.1)	0.5068	1.3 (0.8–2.2)	0.3198
**T3**	1.2 (0.9–1.4)	0.179	1.1 (0.9–1.3)	0.3414	1.1 (0.7–1.7)	0.7205	1.2 (0.7–1.8)	0.5287
**T4**	1.5 (1.2–1.9)	<0.0001	1.5 (1.2–1.9)	<0.0001	2.2 (1.3–3.6)	0.0031	2.3 (1.4–3.8)	0.0016
**Unknown**	1.3 (0.96–1.7)	0.0964	1.3 (1.01–1.7)	0.0455	1.8 (0.9–3.9)	0.1166	2.1 (0.99–4.3)	0.0544
**N stage**								
**N0**	1		1		1		1	
**N1**	1.5 (1.3–1.7)	<0.0001	1.5 (1.3–1.7)	<0.0001	1.3 (1.02–1.7)	0.0347	1.3 (1.04–1.7)	0.0233
**Unknown**	1.3 (0.994–1.6)	0.056	1.3 (1.01–1.6)	0.0374	0.9 (0.5–1.5)	0.6827	0.9 (0.6–1.5)	0.724
**Surgery**								
**No**	1		1		1		1	
**Yes**	0.4 (0.3–0.4)	<0.0001	0.4 (0.3–0.4)	<0.0001	0.5 (0.4–0.7)	0.0002	0.5 (0.4–0.8)	0.0007
**Systemic Therapy**								
**No**	1		1		1		1	
**Yes**	0.7 (0.6–0.8)	<0.0001	0.7 (0.6–0.8)	<0.0001	0.6 (0.5–0.8)	0.0004	0.6 (0.5–0.8)	0.0005
**Radiotherapy**								
**No**	1		1					
**Yes**	1.1 (0.997–1.3)	0.0556						
**Period**								
**2010–2015**	1		1		1		1	
**2016–2020**	0.6 (0.5–0.6)	<0.0001	0.6 (0.5–0.7)	<0.0001	0.8 (0.6–1.02)	0.0685	0.8 (0.6–1.001)	0.0508

Variables that were not significantly associated with survival outcomes (*p* ≥ 0.1) were excluded from the final models. Consequently, no estimates are reported for these variables in the table.

## Data Availability

All data used in this study were obtained from the publicly available SEER Program database (https://seer.cancer.gov/data/, accessed in July 12, 2023).

## Supplementary Materials

The following supporting information can be downloaded at: https://www.mdpi.com/article/10.3390/cancers18172884/s1, Table S1: Distribution of case number by ICD-O-3 and histological type in patients with metastatic sarcomatoid RCC diagnosed during 2010–2020; Table S2: Three-year cancer specific survival and overall survival rates by race and study period among patients diagnosed during 2010–2020; Table S3: Multivariable survival analysis of factors correlated with cancer-specific and overall survival in patients with metastatic sarcomatoid RCC diagnosed during 2016–2017; Table S4: Factors associated with cancer-specific survival in patients with msRCC diagnosed during 2010–2020; Table S5: Factors associated with overall survival in patients with msRCC diagnosed during 2010–2020; Table S6: Three-year cancer specific and overall survival rates by race and histological types among patients msRCC diagnosed during 2010–2020; Figure S1: Flowchart of patient selection from the SEER database (2000–2020); Figure S2: Cumulative incidence of cancer-specific mortality by racial group among patients with metastatic sarcomatoid renal cell carcinoma diagnosed during 2010–2020; Figure S3: Cumulative incidence of cancer-specific mortality by racial group among patients with metastatic sarcomatoid renal cell carcinoma diagnosed during 2010–2015; Figure S4: Cumulative incidence of cancer-specific mortality by racial group among patients with metastatic sarcomatoid renal cell carcinoma diagnosed during 2016–2020; Figure S5: Cumulative incidence of cancer-specific mortality by racial group among patients with clear cell metastatic sarcomatoid renal cell carcinoma diagnosed during 2016–2020; Figure S6: Cumulative incidence of cancer-specific mortality by racial group among patients non-clear cell metastatic sarcomatoid renal cell carcinoma diagnosed during 2016–2020.
