# Supplementary material for: Racial Disparities in Survival Outcomes for Metastatic Renal Cell Carcinoma with Sarcomatoid Differentiation: A SEER-Based Retrospective Analysis (2010–2020)
Source: Cancers (Basel). 2026 Sep 6;18(17):2884. doi: 10.3390/cancers18172884 (PMC13565211; doi:10.3390/cancers18172884)
Supplement: Supplementary file 1 [file cancers-18-02884-s001.zip › cancers-4485783-supplementary.pdf]

**Supplementary data**

**Table S1. Distribution of case number by ICD-O-3 and histological type in patients with metastatic sarcomatoid RCC diagnosed during 2010-2020**

| ICD-O-3: Label | Histological type n (%) | | | | |
|---|---|---|---|---|---|
| | Clear cell | Papillary | Chromophobe | Other | Total |
| 8000/3: Neoplasm, malignant | | | | 2 | 2 |
| 8005/3: Malignant tumor, clear cell type | 1 | | | | 1 |
| 8010/3: Carcinoma, NOS | 10 | | | | 10 |
| 8032/3: Spindle cell carcinoma, NOS | | | | 4 | 4 |
| 8033/3: Pseudosarcomatous carcinoma | | | | 54 | 54 |
| 8050/3: Papillary carcinoma, NOS | | 1 | | | 1 |
| 8120/3: Transitional cell carcinoma, NOS | | | | 4 | 4 |
| 8122/3: Transitional cell carcinoma, spindle cell | | | | 4 | 4 |
| 8130/3: Papillary transitional cell carcinoma | | | | 2 | 2 |
| 8140/3: Adenocarcinoma, NOS | 3 | | | | 3 |
| 8255/3: Adenocarcinoma with mixed subtypes | | | | 212 | 212 |
| 8260/3: Papillary adenocarcinoma, NOS | | 56 | | | 56 |
| 8310/3: Clear cell adenocarcinoma, NOS | 825 | | | | 825 |
| 8312/3: Renal cell carcinoma | 358 | | | | 358 |
| 8317/3: Renal cell carcinoma, chromophobe type | | | 25 | | 25 |
| 8318/3: Renal cell carcinoma, sarcomatoid | 537 | | | | 537 |
| 8319/3: Collecting duct carcinoma | | | | 15 | 15 |
| 8323/3: Mixed cell adenocarcinoma | 6 | | | | 6 |
| 8480/3: Mucinous adenocarcinoma | | | | 1 | 1 |
| 8510/3: Medullary carcinoma, NOS | | | | 2 | 2 |
| Total n (%) | 1740 (82) | 57 (2.7) | 25 (1.2) | 300 (14.1) | 2122 |

**Table S2. Three-year cancer specific survival and overall survival rates by race and study period among patients diagnosed during 2010-2020.**

| | Cancer specific survival | | | Overall survival | | |
|---|---|---|---|---|---|---|
| | 3-year survival rate (%± SE) | Univariable Cox regression | | 3-year survival rate (%± SE) | Univariable Cox regression | |
| | | HR (95% CI) | P value | | HR (95% CI) | P value |
| **Race** | | | | | | |
| **2010-2020** | | | | | | |
| **NHW** | 23.3 ± 1.3 | 1 | | 20.9 ± 1.2 | 1 | |
| **NHB** | 9.3 ± 2.6 | 1.6 (1.3 - 1.9) | <.0001 | 8.6 ± 2.5 | 1.5 (1.3 - 1.8) | <.0001 |
| **Hispanic** | 22.1 ± 2.6 | 1.1 (0.9 - 1.2) | 0.3116 | 19.0 ± 2.3 | 1.1 (1.0 - 1.2) | 0.1851 |
| **API** | 21.2 ± 4.4 | 1.1 (0.8 - 1.3) | 0.6708 | 19.4 ± 4.1 | 1.0 (0.8 - 1.3) | 0.8239 |
| **AIAN** | 23.4 ± 8.8 | 0.8 (0.5 - 1.2) | 0.2989 | 20.7 ± 8.0 | 0.8 (0.6 - 1.3) | 0.4076 |
| **2010-2015** | | | | | | |
| **NHW** | 17.4 ± 1.4 | 1 | | 15.6 ± 1.3 | 1 | |
| **NHB** | 3.8 ± 2.1 | 1.6 (1.3 - 2.0) | <.0001 | 3.5 ± 2.0 | 1.6 (1.2 - 1.9) | 0.0002 |
| **Hispanic** | 16.2 ± 3.1 | 1.1 (0.9 - 1.3) | 0.4491 | 13.8 ± 2.8 | 1.1 (0.9 - 1.3) | 0.3974 |
| **API** | 12.7 ± 4.4 | 1.1 (0.8 - 1.4) | 0.5981 | 11.2 ± 4.0 | 1.1 (0.8 - 1.4) | 0.6625 |
| **AIAN** | 26.7 ± 11.4 | 0.6 (0.4 - 1.2) | 0.1418 | 26.7 ± 11.4 | 0.7 (0.4 - 1.2) | 0.1409 |
| **2016-2020** | | | | | | |
| **NHW** | 31.6 ± 2.2 | 1 | | 28.4 ± 2.0 | 1 | |
| **NHB** | 18.1 ± 5.4 | 1.6 (1.2 - 2.1) | 0.0011 | 16.9 ± 5.1 | 1.5 (1.2 - 2.0) | 0.002 |
| **Hispanic** | 28.4 ± 4.1 | 1.1 (0.9 - 1.4) | 0.2613 | 24.9 ± 3.8 | 1.2 (0.9 - 1.4) | 0.151 |
| **API** | 38.3 ± 8.2 | 0.9 (0.6 - 1.4) | 0.7911 | 36.5 ± 7.9 | 0.9 (0.6 - 1.3) | 0.6502 |
| **AIAN** | 0 | 1.1 (0.5 - 2.0) | 0.8857 | 0 | 1.2 (0.6 - 2.1) | 0.6218 |

AIAN, Non-Hispanic American Indian and Alaska Native (AIAN); API, Non-Hispanic Asian or Pacific Islanders; NHB, Non-Hispanic black; NHW, Non-Hispanic white; SE, standard error.

**Table S3. Multivariable survival analysis of factors correlated with cancer-specific and overall survival in patients with metastatic sarcomatoid RCC diagnosed during 2016-2017.**

| Variable | Cancer-specific survival | | Overall survival | |
|---|---|---|---|---|
| | HR (95% CI) | *P* value | HR (95% CI) | *P* value |
| **Age (years)** | | | | |
| **Groups** | | | | |
| **< 50** | 1 | | 1 | |
| **50-59** | 0.8 (0.6 - 1.2) | 0.3432 | 0.8 (0.5 - 1.1) | 0.1571 |
| **60-69** | 1.1 (0.8 - 1.6) | 0.4482 | 1.1 (0.8 - 1.5) | 0.6109 |
| **70-79** | 0.9 (0.6 - 1.3) | 0.4525 | 0.9 (0.6 - 1.3) | 0.4515 |
| **≥ 80** | 2.3 (1.3 - 4.2) | 0.0045 | 2.9 (1.7 - 4.8) | <.0001 |
| **Race** | | | | |
| **Hispanic** | 1.0 (0.8 - 1.4) | 0.8885 | 1.1 (0.8 - 1.4) | 0.6443 |
| **Non-Hispanic American Indian and Alaska Native** | 1.2 (0.5 - 3.1) | 0.6522 | 1.4 (0.6 - 3.3) | 0.407 |
| **Non-Hispanic Asian and Pacific Islander** | 1.0 (0.6 - 1.7) | 0.9676 | 0.9 (0.6 - 1.5) | 0.7738 |
| **Non-Hispanic Black** | 1.7 (1.1 - 2.6) | 0.0091 | 1.6 (1.1 - 2.4) | 0.0224 |
| **Non-Hispanic White** | 1 | | 1 | |
| | | | | |
| **Region** | | | | |
| **South** | 1 | | 1 | |
| **West** | 0.9 (0.6 - 1.4) | 0.5708 | 0.9 (0.6 - 1.4) | 0.7337 |
| **Midwest** | 0.7 (0.5 - 1.2) | 0.2079 | 0.8 (0.5 - 1.2) | 0.2872 |
| **Northeast** | 0.5 (0.3 - 0.9) | 0.0222 | 0.6 (0.3 - 0.9) | 0.0293 |
| **T stage** | | | | |
| **T1** | 1 | | 1 | |
| **T2** | 1.5 (0.91 - 2.32) | 0.1196 | 1.5 (0.91 - 2.32) | 0.1196 |
| **T3** | 1.2 (0.80 - 1.76) | 0.3932 | 1.2 (0.80 - 1.76) | 0.3932 |
| **T4** | 1.8 (1.21 - 2.79) | 0.0046 | 1.8 (1.21 - 2.79) | 0.0046 |
| **Unknown** | 1.8 (1.02 - 3.22) | 0.0421 | 1.8 (1.02 - 3.22) | 0.0421 |
| **N stage** | | | | |
| **N0** | 1 | | 1 | |
| **N1** | 0.3 (0.2 - 0.4) | <.0001 | 1.4 (1.10 - 1.72) | 0.006 |
| **Unknown** | 0.3 (0.2 - 0.4) | <.0001 | 1.3 (0.84 - 1.95) | 0.2598 |
| **Surgery** | | | | |
| **No** | 1 | | 1 | |
| **Yes** | 0.3 (0.2 - 0.4) | <.0001 | 0.3 (0.22 - 0.40) | <.0001 |
| **Systemic Therapy** | | | | |
| **No** | 1 | | 1 | |
| **Yes** | 0.8 (0.6 - 1.0) | 0.0172 | 0.7 (0.61 - 0.93) | 0.0074 |

**Table S4. Factors associated with cancer-specific survival in patients with msRCC diagnosed during 2010-2020.**

| | Mode I | | Model II | | Model III | | Model IV | |
|---|---|---|---|---|---|---|---|---|
| **Variable** | HR (95% CI) | *P* value | HR (95% CI) | *P* value | HR (95% CI) | *P* value | HR (95% CI) | *P* value |
| **Race** | | | | | | | | |
| **Hispanic** | 1.1 (0.9 - 1.2) | 0.3116 | 1.1 (0.95 - 1.3) | 0.1961 | 1.1 (0.95 - 1.3) | 0.1908 | 1.1 (0.9 - 1.3) | 0.2309 |
| **Non-Hispanic American Indian and Alaska Native** | 0.8 (0.5 - 1.2) | 0.2989 | 0.8 (0.5 - 1.2) | 0.2295 | 0.9 (0.6 - 1.4) | 0.5367 | 0.8 (0.5 - 1.3) | 0.4015 |
| **Non-Hispanic Asian and Pacific Islander** | 1.1 (0.8 - 1.3) | 0.6708 | 1.1 (0.8 - 1.3) | 0.6524 | 1.1 (0.9 - 1.4) | 0.4906 | 1.1 (0.9 - 1.4) | 0.4555 |
| **Non-Hispanic Black** | 1.6 (1.3 - 1.9) | <.0001 | 1.7 (1.4 - 2.0) | <.0001 | 1.6 (1.3 - 1.9) | <.0001 | 1.5 (1.3 - 1.9) | <.0001 |
| **Non-Hispanic White** | 1 | | 1 | | 1 | | 1 | |
| **Age (years)** | | | | | | | | |
| **Groups** | | | | | | | | |
| **< 50** | | | 1 | | 1 | | 1 | |
| **50-59** | | | 0.9 (0.8 - 1.1) | 0.2477 | 0.9 (0.8 - 1.1) | 0.2054 | 0.8 (0.7 – 0.997) | 0.0462 |
| **60-69** | | | 1.0 (0.8 - 1.2) | 0.9673 | 1.0 (0.9 - 1.2) | 0.6418 | 1.0 (0.8 - 1.1) | 0.6764 |
| **70-79** | | | 1.1 (0.9 - 1.3) | 0.2737 | 1.2 (0.99 - 1.4) | 0.0689 | 1.1 (0.9 - 1.3) | 0.5511 |
| **≥ 80** | | | 2.2 (1.7 - 2.8) | <.0001 | 0.9 (0.8 - 1.1) | 0.2054 | 1.7 (1.3 - 2.3) | <.0001 |
| **Gender** | | | | | | | | |
| **Male** | | | 1 | | 1 | | 1 | |
| **Female** | | | 0.9 (0.8 - 1.1) | 0.3332 | 1.0 (0.8 - 1.1) | 0.3878 | 1.0 (0.9 - 1.1) | 0.5395 |
| **Marital status** | | | | | | | | |
| **Married** | | | 1 | | 1 | | 1 | |
| **Unmarried** | | | 1.1 (0.995 - 1.2) | 0.0604 | 1.1 (0.997 - 1.2) | 0.0561 | 1.1 (0.96 - 1.2) | 0.2398 |
| **Unknown** | | | 1.0 (0.7 - 1.4) | 0.98 | 1.1 (0.8 - 1.4) | 0.7521 | 1.0 (0.8 - 1.4) | 0.7775 |
| **Region** | | | | | | | | |
| **West** | | | 1 | | 1 | | 1 | |
| **South** | | | 0.8 (0.7 – 0.98) | 0.0245 | 0.9 (0.7 – 0.99) | 0.0414 | 0.9 (0.7 - 1.003) | 0.0541 |
| **Midwest** | | | 1.1 (0.9 - 1.4) | 0.405 | 1.1 (0.9 - 1.4) | 0.3581 | 1.0 (0.8 - 1.3) | 0.7116 |
| **Northeast** | | | 0.9 (0.8 - 1.1) | 0.2811 | 0.9 (0.7 - 1.02) | 0.0797 | 0.9 (0.7 - 1.04) | 0.1266 |

| | | | | | | | | |
|---|---|---|---|---|---|---|---|---|
| **Income ($)** | | | | | | | | |
| **Less than 60,000** | | | 1 | | 1 | | 1 | |
| **60,000-74,999** | | | 0.9 (0.8 - 1.04) | 0.1602 | 0.9 (0.8 - 1.1) | 0.3322 | 0.9 (0.8 - 1.1) | 0.3751 |
| **75,000 and over** | | | 0.8 (0.7 – 0.98) | 0.0243 | 0.8 (0.7 – 0.97 | 0.0186 | 0.8 (0.7 – 0.98) | 0.0283 |
| **Period** | | | | | | | | |
| **2010-2015** | | | 1 | | 1 | | 1 | |
| **2016-2020** | | | 0.7 (0.6 - 0.8) | <.0001 | 0.7 (0.6 - 0.7) | <.0001 | 0.6 (0.6 - 0.7) | <.0001 |
| **T stage** | | | | | | | | |
| **T1** | | | | | 1 | | 1 | |
| **T2** | | | | | 1.0 (0.8 - 1.3) | 0.9471 | 1.1 (0.9 - 1.4) | 0.4225 |
| **T3** | | | | | 0.8 (0.7 - 0.9) | 0.0085 | 1.1 (0.9 - 1.3) | 0.2962 |
| **T4** | | | | | 1.3 (1.04 - 1.6) | 0.0176 | 1.6 (1.3 - 2.0) | <.0001 |
| **Unknown** | | | | | 1.8 (1.4 - 2.4) | <.0001 | 1.4 (1.04 - 1.9) | 0.0242 |
| **N stage** | | | | | | | | |
| **N0** | | | | | 1 | | 1 | |
| **N1** | | | | | 1.6 (1.4 - 1.8) | <.0001 | 1.5 (1.4 - 1.7) | <.0001 |
| **Unknown** | | | | | 1.4 (1.1 - 1.7) | 0.0071 | 1.1 (0.9 - 1.4) | 0.2996 |
| **Grade** | | | | | | | | |
| **I** | | | | | 1.4 (0.7 - 3.0) | 0.3737 | 1.1 (0.5 - 2.4) | 0.7982 |
| **II** | | | | | 1 | | 1 | |
| **III/IV** | | | | | 1.0 (0.7 - 1.5) | 0.9155 | 1.1 (0.8 - 1.6) | 0.5981 |
| **Unknown** | | | | | 1.1 (0.8 - 1.6) | 0.5800 | 0.9 (0.6 - 1.3) | 0.5985 |
| **Histology** | | | | | | | | |
| **Clear-cell** | | | | | 1 | | | |
| **Papillary** | | | | | 0.8 (0.6 - 1.1) | 0.2656 | 0.9 (0.6 - 1.2) | 0.3979 |
| **Chromophobe** | | | | | 1.4 (0.9 - 2.3) | 0.1604 | 1.7 (1.1 - 2.8) | 0.0228 |
| **Other** | | | | | 1.2 (1.02 - 1.4) | 0.0232 | 1.2 (1.1 - 1.4) | 0.0074 |
| **Tumor size (cm)** | | | | | | | | |
| **≤3** | | | | | 1 | | 1 | |
| **>3** | | | | | 1.0 (0.7 - 1.3) | 0.8364 | 1.1 (0.8 - 1.4) | 0.7467 |
| **Unknown** | | | | | 1.0 (0.7 - 1.4) | 0.9116 | 1.0 (0.7 - 1.5) | 0.9855 |
| **Surgery** | | | | | | | | |
| **No** | | | | | | | 1 | |

| | | | | | | | | |
|---|---|---|---|---|---|---|---|---|
| **Yes** | | | | | | | 0.4 (0.3 - 0.4) | <.0001 |
| **Systemic Therapy** | | | | | | | | |
| **No** | | | | | | | 1 | |
| **Yes** | | | | | | | 0.7 (0.6 - 0.7) | <.0001 |
| **Radiotherapy** | | | | | | | | |
| **No** | | | | | | | 1 | |
| **Yes** | | | | | | | 1.2 (1.1 - 1.3) | 0.0048 |

**Table S5. Factors associated with overall survival in patients with msRCC diagnosed during 2010-2020.**

| | Mode I | | Model II | | Model III | | Model IV | |
|---|---|---|---|---|---|---|---|---|
| **Variable** | HR (95% CI) | *P* value | HR (95% CI) | *P* value | HR (95% CI) | *P* value | HR (95% CI) | *P* value |
| **Race** | | | | | | | | |
| **Hispanic** | 1.1 (0.96 - 1.2) | 0.1851 | 1.1 (0.98 - 1.3) | 0.0835 | 1.1 (1.0 - 1.3) | 0.0856 | 1.1 (0.98 - 1.3) | 0.106 |
| **Non-Hispanic American Indian and Alaska Native** | 0.8 (0.6 - 1.3) | 0.4076 | 0.8 (0.5 - 1.2) | 0.3609 | 0.9 (0.6 - 1.4) | 0.7956 | 0.9 (0.6 - 1.4) | 0.6118 |
| **Non-Hispanic Asian and Pacific Islander** | 1.0 (0.8 - 1.3) | 0.8239 | 1.0 (0.8 - 1.3) | 0.6783 | 1.1 (0.9 - 1.3) | 0.5331 | 1.1 (0.9 - 1.4) | 0.4756 |
| **Non-Hispanic Black** | 1.5 (1.3 - 1.8) | <.0001 | 1.6 (1.3 - 1.9) | <.0001 | 1.5 (1.3 - 1.8) | <.0001 | 1.5 (1.2 - 1.8) | <.0001 |
| **Non-Hispanic White** | 1 | | 1 | | 1 | | 1 | |
| **Age (years)** | | | | | | | | |
| **Groups** | | | | | | | | |
| **< 50** | | | 1 | | 1 | | 1 | |
| **50-59** | | | 0.9 (0.7 - 1.04) | 0.1306 | 0.9 (0.7 - 1.03) | 0.1056 | 0.8 (0.7 – 0.96) | 0.0155 |
| **60-69** | | | 1.0 (0.9 - 1.2) | 0.9072 | 1.1 (0.9 - 1.3) | 0.4915 | 1.0 (0.8 - 1.2) | 0.7739 |
| **70-79** | | | 1.1 (0.9 - 1.3) | 0.2481 | 1.2 (0.998 - 1.4) | 0.0526 | 1.1 (0.9 - 1.3) | 0.5302 |
| **≥ 80** | | | 2.5 (1.9 - 3.1) | <.0001 | 2.3 (1.8 - 3.0) | <.0001 | 1.9 (1.5 - 2.5) | <.0001 |
| **Gender** | | | | | | | | |
| **Male** | | | 1 | | 1 | | 1 | |
| **Female** | | | 0.9 (0.8 - 1.03) | 0.1614 | 0.9 (0.8 - 1.04) | 0.2076 | 0.9 (0.8 - 1.1) | 0.3239 |
| **Marital status** | | | | | | | | |
| **Married** | | | 1 | | 1 | | 1 | |
| **Unmarried** | | | 1.1 (1.01 - 1.3) | 0.0261 | 1.1 (1.02 - 1.3) | 0.0223 | 1.1 (0.98 - 1.2) | 0.1321 |
| **Unknown** | | | 0.9 (0.7 - 1.3) | 0.7274 | 1.0 (0.7 - 1.3) | 0.9887 | 1.0 (0.7 - 1.3) | 0.9742 |
| **Region** | | | | | | | | |
| **West** | | | 1 | | 1 | | 1 | |
| **South** | | | 0.9 (0.8 – 0.99) | 0.0345 | 0.9 (0.8 - 1.01) | 0.0635 | 0.9 (0.8 - 1.02) | 0.0832 |
| **Midwest** | | | 1.1 (0.9 - 1.4) | 0.5015 | 1.1 (0.9 - 1.4) | 0.4282 | 1.0 (0.8 - 1.3) | 0.8444 |
| **Northeast** | | | 0.9 (0.8 - 1.1) | 0.4405 | 0.9 (0.8 - 1.04) | 0.1242 | 0.9 (0.8 - 1.1) | 0.2049 |
| **Income ($)** | | | | | | | | |

| | | | | | | | | |
|---|---|---|---|---|---|---|---|---|
| **Less than 60,000** | | | 1 | | 1 | | 1 | |
| **60,000-74,999** | | | 0.9 (0.8 - 1.1) | 0.2393 | 1.0 (0.8 - 1.1) | 0.5036 | 1.0 (0.8 - 1.1) | 0.5487 |
| **75,000 and over** | | | 0.8 (0.7 – 0.95) | 0.0098 | 0.8 (0.7 - 0.9) | 0.0079 | 0.8 (0.7 – 0.96) | 0.0128 |
| **Period** | | | | | | | | |
| **2010-2015** | | | 1 | | 1 | | 1 | |
| **2016-2020** | | | 0.7 (0.6 - 0.8) | <.0001 | 0.7 (0.6 - 0.8) | <.0001 | 0.7 (0.6 - 0.7) | <.0001 |
| **T stage** | | | | | | | | |
| **T1** | | | | | 1 | | 1 | |
| **T2** | | | | | 1.0 (0.8 - 1.3) | 0.739 | 1.1 (0.9 - 1.4) | 0.3091 |
| **T3** | | | | | 0.8 (0.7 - 0.9) | 0.0066 | 1.1 (0.9 - 1.3) | 0.281 |
| **T4** | | | | | 1.3 (1.1 - 1.6) | 0.0063 | 1.7 (1.4 - 2.0) | <.0001 |
| **Unknown** | | | | | 1.9 (1.4 - 2.5) | <.0001 | 1.4 (1.1 - 1.9) | 0.0086 |
| **N stage** | | | | | | | | |
| **N0** | | | | | 1 | | 1 | |
| **N1** | | | | | 1.6 (1.5 - 1.8) | <.0001 | 1.5 (1.4 - 1.7) | <.0001 |
| **Unknown** | | | | | 1.4 (1.1 - 1.7) | 0.0023 | 1.2 (0.9 - 1.4) | 0.1814 |
| **Grade** | | | | | | | | |
| **I** | | | | | 1.2 (0.6 - 2.6) | 0.5945 | 1.0 (0.5 - 2.0) | 0.9087 |
| **II** | | | | | 1 | | 1 | |
| **III/IV** | | | | | 1.0 (0.7 - 1.4) | 0.881 | 1.1 (0.7 - 1.5) | 0.7413 |
| **Unknown** | | | | | 1.1 (0.7 - 1.5) | 0.7241 | 0.9 (0.6 - 1.2) | 0.4353 |
| **Histology** | | | | | | | | |
| **Clear-cell** | | | | | 1 | | | |
| **Papillary** | | | | | 0.8 (0.6 - 1.1) | 0.2465 | 0.9 (0.7 - 1.2) | 0.3899 |
| **Chromophobe** | | | | | 1.5 (0.9 - 2.3) | 0.0973 | 1.8 (1.1 - 2.8) | 0.0104 |
| **Other** | | | | | 1.1 (0.999 - 1.3) | 0.0522 | 1.2 (1.0 - 1.3) | 0.0215 |
| **Tumor size (cm)** | | | | | | | | |
| **≤3** | | | | | 1 | | 1 | |
| **>3** | | | | | 0.9 (0.7 - 1.3) | 0.7109 | 1.0 (0.8 - 1.4) | 0.8596 |
| **Unknown** | | | | | 1.0 (0.7 - 1.4) | 0.9887 | 1.0 (0.7 - 1.5) | 0.9251 |
| **Surgery** | | | | | | | | |
| **No** | | | | | | | 1 | |
| **Yes** | | | | | | | 0.4 (0.3 - 0.4) | <.0001 |

| | | | | | | | | |
|---|---|---|---|---|---|---|---|---|
| **Systemic Therapy** | | | | | | | | |
| **No** | | | | | | | 1 | |
| **Yes** | | | | | | | 0.7 (0.6 - 0.7) | <.0001 |
| **Radiotherapy** | | | | | | | | |
| **No** | | | | | | | 1 | |
| **Yes** | | | | | | | 1.1 (1.02 - 1.3) | 0.0207 |

**Table S6. Three-year cancer specific and overall survival rates by race and histological types among patients msRCC diagnosed during 2010-2020**

| | Cancer specific survival | | | Overall survival | | |
|---|---|---|---|---|---|---|
| | 3-year survival rate (%± SE) | Univariable Cox regression | | 3-year survival rate (%± SE) | Univariable Cox regression | |
| | | HR (95% CI) | P value | | HR (95% CI) | P value |
| **Race** | | | | | | |
| **Histological type** | | | | | | |
| **CC** | | | | | | |
| **NHW** | 24.9 ± 1.5 | 1 | | 22.3 ± 1.4 | 1 | |
| **NHB** | 11.4 ± 3.2 | 1.5 (1.2 - 1.9) | <.0001 | 10.8 ± 3.0 | 1.4 (1.2 - 1.8) | 0.0003 |
| **Hispanic** | 21.8 ± 2.8 | 1.1 (1.0 - 1.3) | 0.1118 | 19.2 ± 2.6 | 1.1 (1.0 - 1.3) | 0.0835 |
| **API** | 24.1 ± 5.1 | 1.0 (0.8 - 1.3) | 0.7732 | 22.3 ± 4.9 | 1.0 (0.8 - 1.3) | 0.9621 |
| **AIAN** | 20.7 ± 8.9 | 0.9 (0.6 - 1.4) | 0.5856 | 18.2 ± 8.0 | 0.9 (0.6 - 1.4) | 0.6078 |
| **Non-CC** | | | | | | |
| **NHW** | 17.0 ± 2.4 | 1 | | 15.5 ± 2.3 | 1 | |
| **NHB** | 0 | 2.0 (1.3 - 2.8) | 0.0005 | 0 | 1.9 (1.3 - 2.8) | 0.0004 |
| **Hispanic** | 23.6 ± 6.3 | 0.9 (0.6 - 1.2) | 0.4656 | 19.1 ± 5.7 | 0.9 (0.7 - 1.3) | 0.6439 |
| **API** | 10.7 ± 7.1 | 1.1 (0.7 - 1.8) | 0.6981 | 9.5 ± 6.4 | 1.1 (0.7 - 1.8) | 0.6603 |
| **AIAN** | 50.0 ± 35.4 | 0.3 (0.0 - 2.3) | 0.267 | 50.0 ± 35.4 | 0.6 (0.1 - 2.3) | 0.4375 |

AIAN, Non-Hispanic American Indian and Alaska Native (AIAN); API, Non-Hispanic Asian or Pacific Islanders; CC, clear-cell; Non-CC, non-clear cell; NHB, Non-Hispanic black; NHW, Non-Hispanic white; SE, standard error.

**Figure S1. Flowchart of patient selection from the SEER database (2000-2020).**

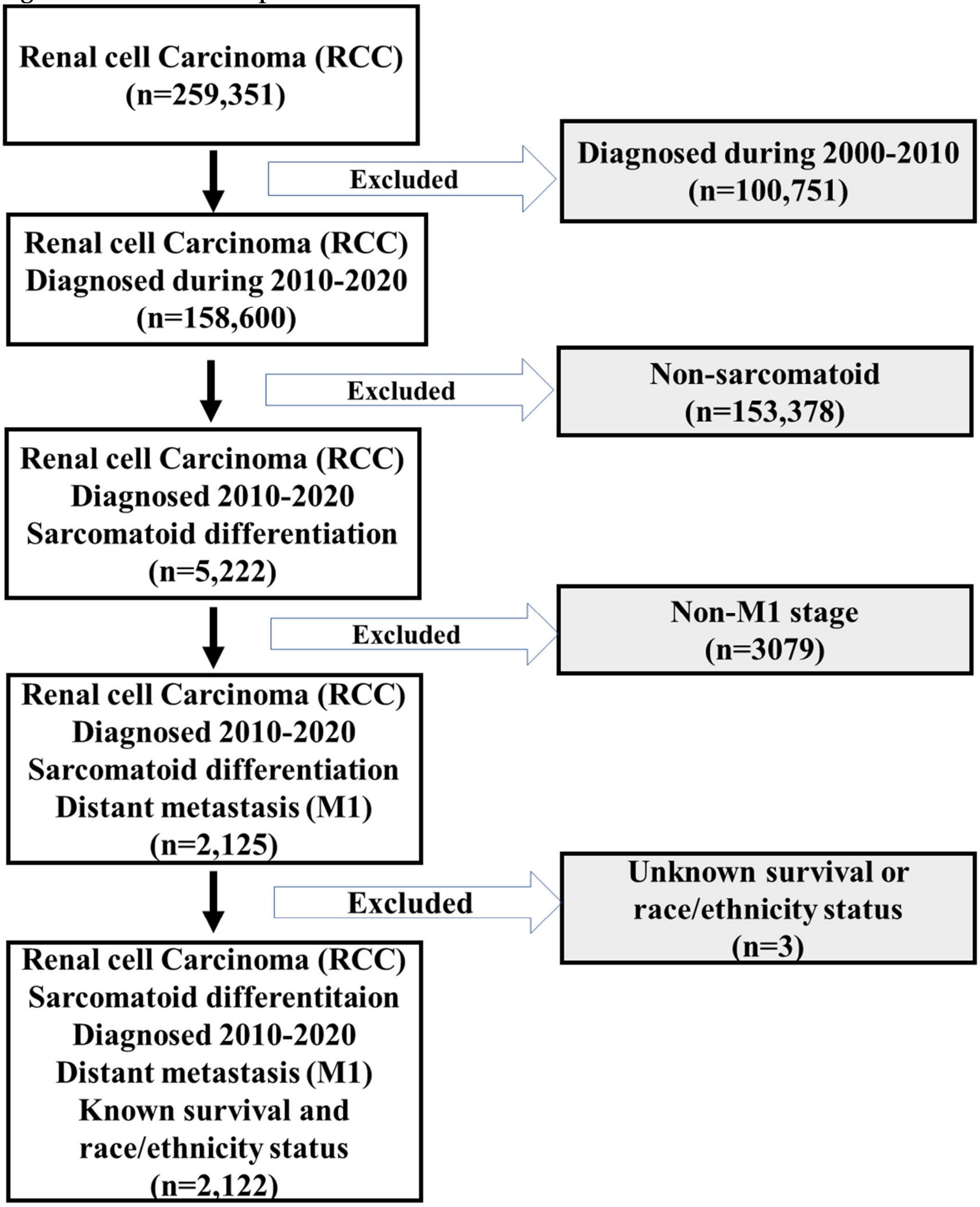


Among the 153,378 patients excluded because sarcomatoid differentiation was not confirmed, 110,981 were classified as "sarcomatoid differentiation not present/not identified," 40,104 as "not documented/assessed or unknown whether assessed; no surgical resection of the primary tumor," 2,086 had blank values, and 207 as "not applicable: not a renal cell carcinoma morphology."

**Figure S2. Cumulative incidence of cancer-specific mortality by racial group among patients with metastatic sarcomatoid renal cell carcinoma diagnosed during 2010-2020.**

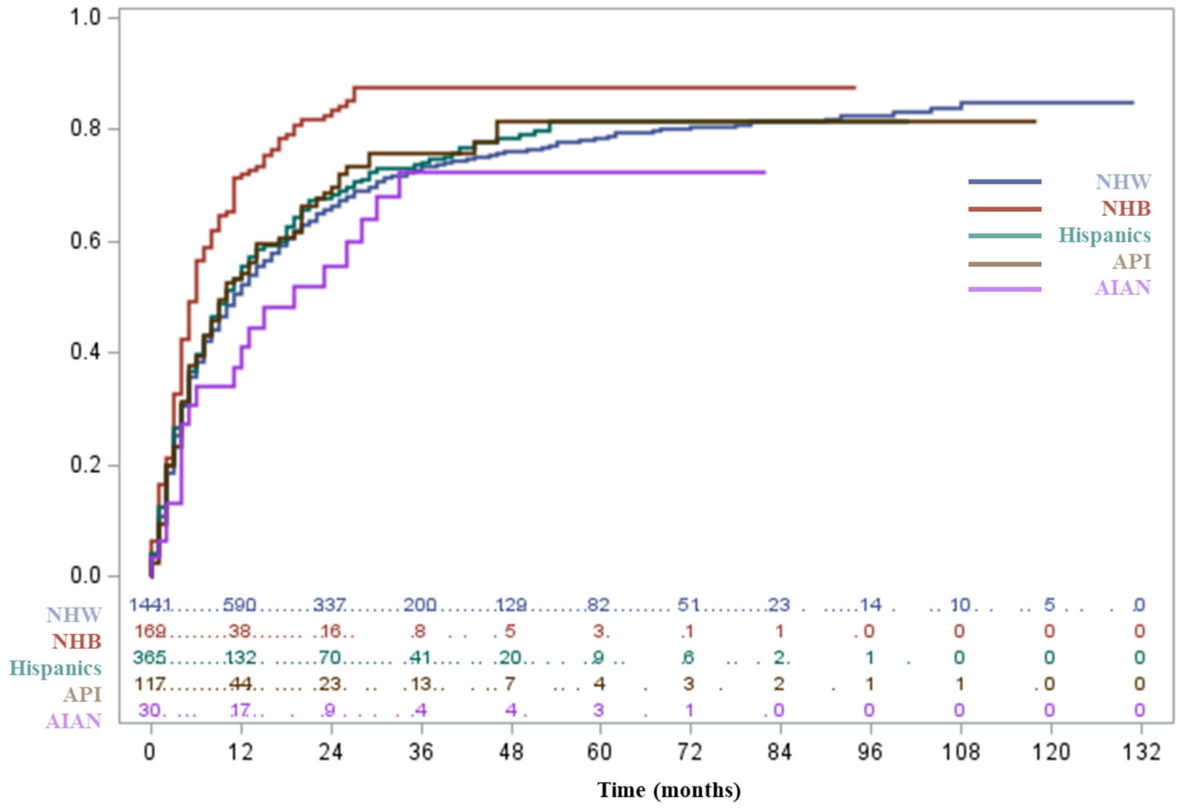


AIAN, Non-Hispanic American Indian and Alaska Native; API, Non-Hispanic Asian or Pacific Islanders; NHB, Non-Hispanic Black; NHW, Non-Hispanic White.

**Figure S3. Cumulative incidence of cancer-specific mortality by racial group among patients with metastatic sarcomatoid renal cell carcinoma diagnosed during 2010-2015.**

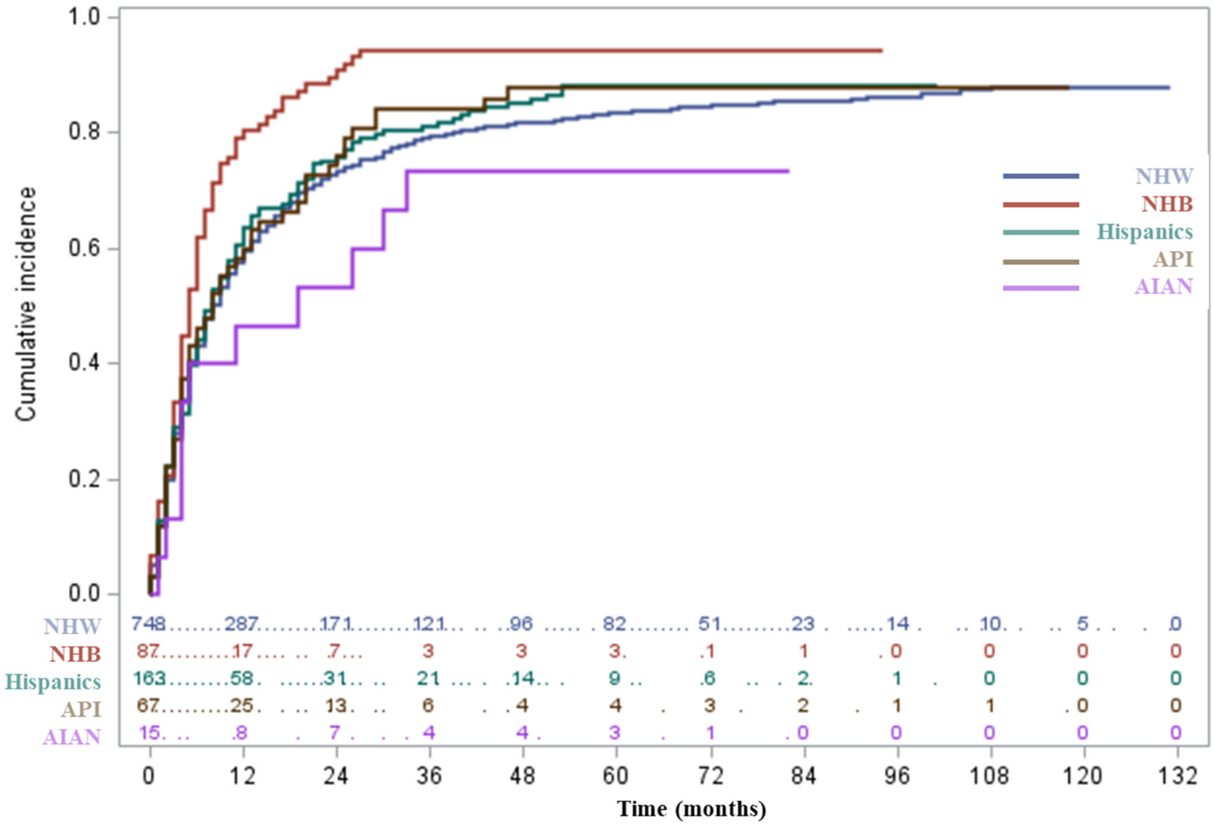


AIAN, Non-Hispanic American Indian and Alaska Native; API, Non-Hispanic Asian or Pacific Islanders; NHB, Non-Hispanic Black; NHW, Non-Hispanic White.

**Figure S4. Cumulative incidence of cancer-specific mortality by racial group among patients with metastatic sarcomatoid renal cell carcinoma diagnosed during 2016-2020.**

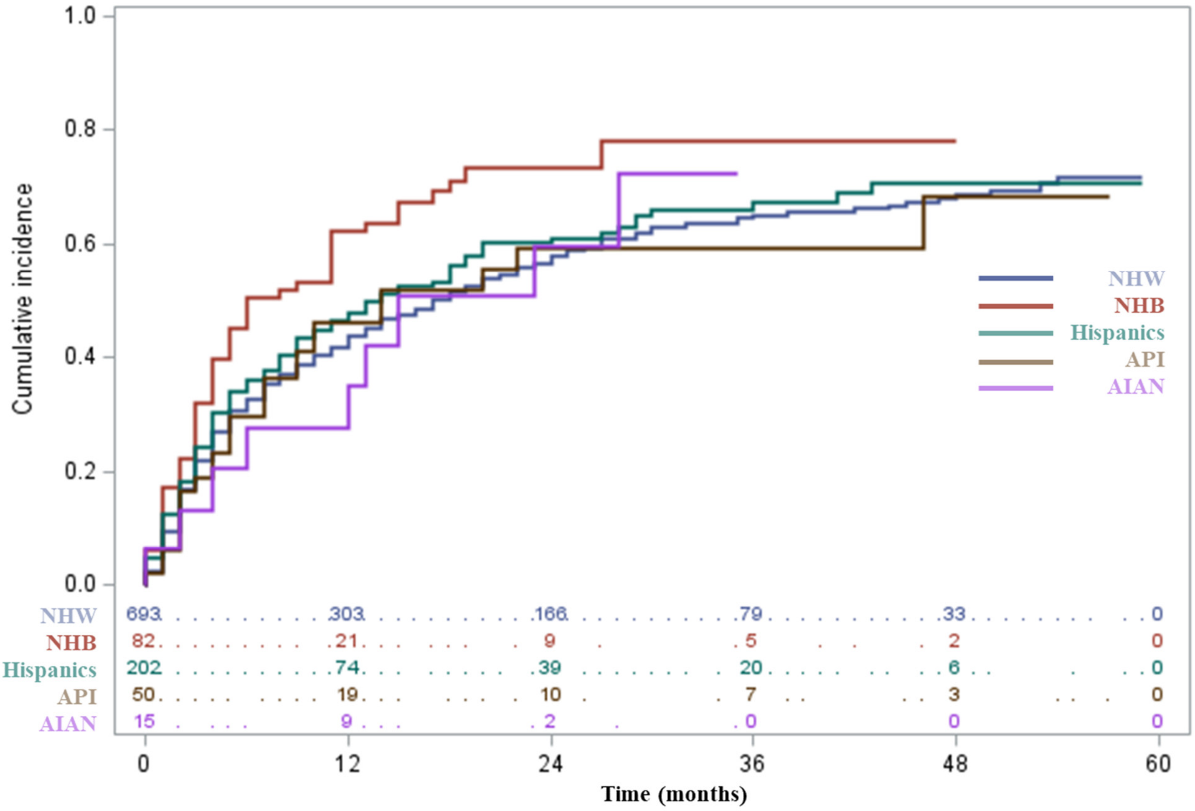


AIAN, Non-Hispanic American Indian and Alaska Native; API, Non-Hispanic Asian or Pacific Islanders; NHB, Non-Hispanic Black; NHW, Non-Hispanic White.

**Figure S5. Cumulative incidence of cancer-specific mortality by racial group among patients with clear-cell metastatic sarcomatoid renal cell carcinoma diagnosed during 2016-2020.**

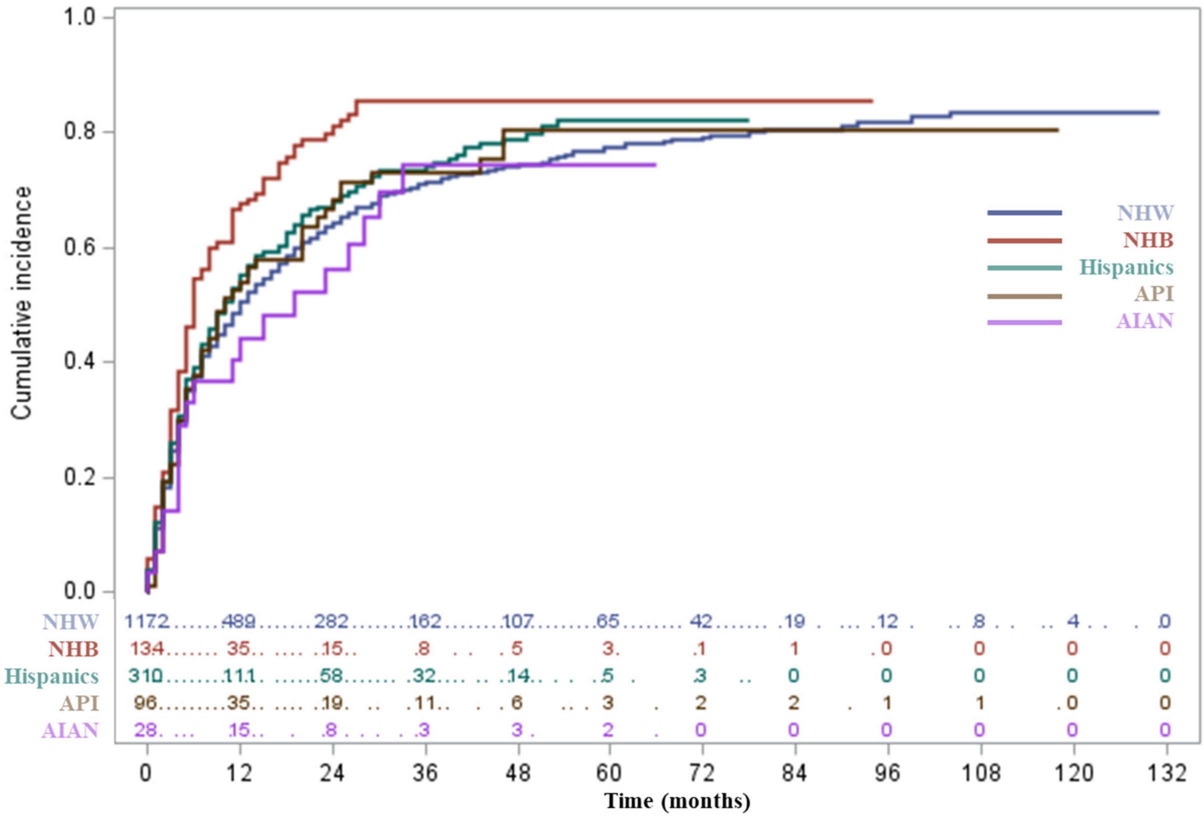


AIAN, Non-Hispanic American Indian and Alaska Native; API, Non-Hispanic Asian or Pacific Islanders; NHB, Non-Hispanic Black; NHW, Non-Hispanic White.

**Figure S6. Cumulative incidence of cancer-specific mortality by racial group among patients non-clear-cell metastatic sarcomatoid renal cell carcinoma diagnosed during 2016-2020.**

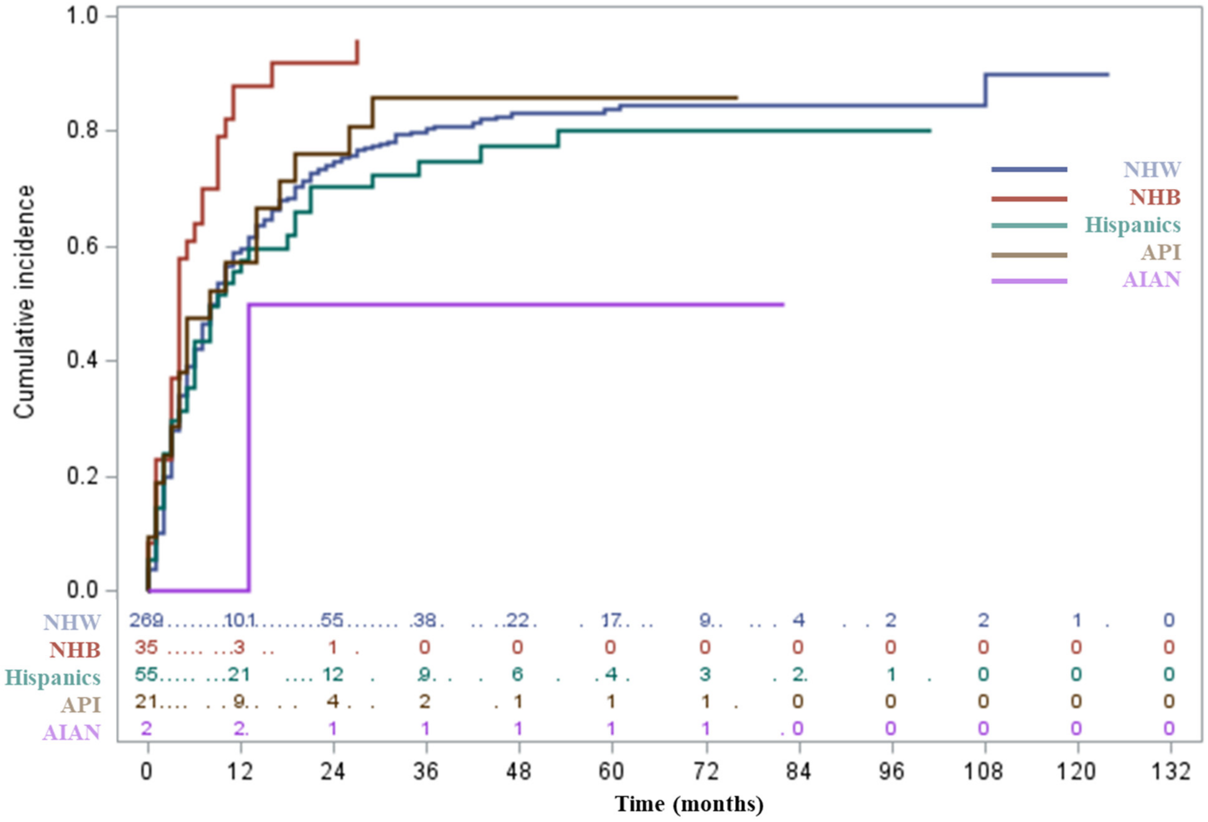


AIAN, Non-Hispanic American Indian and Alaska Native; API, Non-Hispanic Asian or Pacific Islanders; NHB, Non-Hispanic Black; NHW, Non-Hispanic White.
